# Supplementary material for: Digital quantum simulation of Floquet symmetry-protected topological phases
Source: Nature. 2022 Jul 20;607(7919):468–73. doi: 10.1038/s41586-022-04854-3 (PMC9300455; doi:10.1038/s41586-022-04854-3)
Supplement: Supplementary file 1 — This Supplementary Information file contains the following seven sections: I. Theoretical understanding; II. Details of the TEBD method; III. Experimental details; IV. Numerical simulations considering experimental imperfections; V. Quantum-state tomography; VI. Dynamics of entanglement; VII. Extended models. It includes Supplementary Tables 1 and 2, Figs. 1–10 and additional references. [file 41586_2022_4854_MOESM1_ESM.pdf]

nature portfolio

https://doi.org/10.1038/s41586-022-04854-3

Supplementary information

Digital quantum simulation of Floquet symmetry-protected topological phases

In the format provided by the authors and unedited

Supplementary Information: Digital quantum simulation of Floquet symmetry-protected topological phases

Xu Zhang[1,*], Wenjie Jiang[2,*], Jinfeng Deng[1,*], Ke Wang[1], Jiachen Chen[1], Pengfei Zhang[1], Wenhui Ren[1], Hang Dong[1], Shibo Xu[1], Yu Gao[1], Feitong Jin[1], Xuhao Zhu[1], Qiujiang Guo[1,3], Hekang Li[1,3], Chao Song[1,3], Alexey V. Gorshkov[4], Thomas Iadecola[5,6], Fangli Liu[4,7], Zhe-Xuan Gong[8,9], Zhen Wang[1,3,†], Dong-Ling Deng[2,10,‡], and H. Wang[1,3]

[1]*Department of Physics, ZJU-Hangzhou Global Scientific and Technological Innovation Center, Interdisciplinary Center for Quantum Information, and Zhejiang Province Key Laboratory of Quantum Technology and Device, Zhejiang University, Hangzhou 310027, China*
[2] *Center for Quantum Information, IIIS, Tsinghua University, Beijing 100084, China*
[3] *Alibaba-Zhejiang University Joint Research Institute of Frontier Technologies, Hangzhou 310027, China*
[4] *Joint Quantum Institute and Joint Center for Quantum Information and Computer Science, University of Maryland and NIST, College Park, MD, USA*
[5] *Department of Physics and Astronomy, Iowa State University, Ames, Iowa 50011, USA*
[6] *Ames Laboratory, Ames, Iowa 50011, USA*
[7] *QuEra Computing Inc., Boston, Massachusetts 02135, USA*
[8] *Department of Physics, Colorado School of Mines, Golden, Colorado 80401, USA*
[9] *National Institute of Standards and Technology, Boulder, Colorado 80305, USA*
[10] *Shanghai Qi Zhi Institute, 41th Floor, AI Tower, No. 701 Yunjin Road, Xuhui District, Shanghai 200232, China*

Here we provide more details on the theory of the Floquet symmetry-protected topological phase (Sec. I), on our numerical simulations (Sec. II), and on our experimental setup (Sec. III). We also provide additional experimental data (Sec. VI).

I. THEORETICAL UNDERSTANDING

A. Introduction to Floquet time crystals

In order to obtain a better intuitive understanding of the Floquet symmetry-protected topological (FSPT) phase, we first introduce the basic concepts behind Floquet time crystals and present a prototypical model as a concrete example.

Spontaneous symmetry breaking is an important concept in modern physics. It occurs when the steady state of a physical system does not respect the symmetries of the Hamiltonian governing this system. An important example that manifests spontaneous symmetry breaking is an ordinary crystal, which breaks the continuous spatial translation symmetry. More precisely, in a crystal, the state of the system, unlike its Hamiltonian, is not invariant under continuous translation operators. Analogously, systems that spontaneously break time-translation symmetry are named time crystals [S1, S2]. Although there is a no-go theorem for continuous time crystals at equilibrium [S3, S4], Floquet time crystals manifest themselves in many physical systems. There are two equivalent definitions of Floquet time crystals in Ref. [S5], which characterize this concept from the perspective of the expectation value of an operator and from the perspective of the eigenstates of the Floquet evolution unitary, respectively. The first definition states that time-translation symmetry breaking occurs if, for every short-range correlated state $|\psi(t)\rangle$ at arbitrary time t, there exists an operator O satisfying $\langle\psi(t+T)|\, O\, |\psi(t+T)\rangle \neq \langle\psi(t)|\, O\, |\psi(t)\rangle$, where $|\psi(t+T)\rangle = U_F(T)\,|\psi(t)\rangle$, with $U_F(T)$ the Floquet evolution unitary corresponding to one period T. This definition implies how to observe time crystals experimentally and is used in our paper. The second definition states that time-translation symmetry breaking occurs if all eigenstates of the Floquet evolution unitary are long-range correlated. This concept is used in our theoretical analysis.

To be more concrete, we introduce the following prototypical time-dependent Hamiltonian previously studied in Refs. [S5, S6] as an example of a Floquet time crystal:

$$H_F(t) = \begin{cases} H_1 &= \pi/2 \sum_k \hat{\sigma}_k^x, \qquad 0 \le t < T', \\ H_2 &= \sum_k J_k \hat{\sigma}_k^z \hat{\sigma}_{k+1}^z + h_k^z \hat{\sigma}_k^z, \quad T' \le t < T, \end{cases} \tag{S1}$$

where J_k and h_k^z are uniformly chosen from the following intervals: $J_k \in [J/2, 3J/2]$ and $h_k^z \in [0, h^z]$. We set $T = 2T' = 2$. The Floquet evolution operator for one period can then be written as $U_F = \exp(-iH_2)\exp(-i\pi/2\sum_k \hat{\sigma}_k^x)$.

We consider eigenstates of H_2, which are product states in the computational z basis: $|\Theta\rangle = |\{s_k\}\rangle$ with $s_k = \pm 1$. Such states are easy to prepare experimentally. Since U_F has the effect, up to a global phase, of flipping all spins, the state $|\Theta\rangle$ is related to another state $|-\Theta\rangle = |\{-s_k\}\rangle$, which is also an eigenstate of H_2. Defining $E^+(\Theta)$ and $E^-(\Theta)$ via $\sum_k J_k \hat{\sigma}_k^z \hat{\sigma}_{k+1}^z |\Theta\rangle = E^+(\Theta)\,|\Theta\rangle$ and $\sum_k h_k^z \hat{\sigma}_k^z |\Theta\rangle = E^-(\Theta)\,|\Theta\rangle$, we have

$$U_F\,|\Theta\rangle = \exp[-i(E^+(\Theta) - E^-(\Theta))]\,|-\Theta\rangle\,, \tag{S2}$$

$$U_F\,|-\Theta\rangle = \exp[-i(E^+(\Theta) + E^-(\Theta))]\,|\Theta\rangle\,. \tag{S3}$$

Therefore, in the subspace formed by $|\pm\Theta\rangle$, U_F has the matrix form

$$U_F = \begin{bmatrix} 0 & e^{-i(E^+(\Theta)-E^-(\Theta))} \\ e^{-i(E^+(\Theta)+E^-(\Theta))} & 0 \end{bmatrix}. \tag{S4}$$

Diagonalizing this matrix gives eigenvalues $\pm\exp(-iE^{+}(\Theta))$ and eigenstates $|\Theta\rangle \pm \exp[iE^{-}(\Theta)]\,|{-\Theta}\rangle$. The eigenstates of U_F are thus paired cat states with long-range correlations. Thus, this model satisfies the second definition of a Floquet time crystal in Ref. [S5], so discrete time-translation symmetry breaking occurs in this system. (Note that, in order for these correlations to be stable to perturbations, disorder in the couplings J_k and h_k^z that is sufficiently strong to render U_F many-body localized is required.) Futhermore, as the Floquet operator has eigenvalues $\pm\exp(-iE^{+}(\Theta))$, if we diagonalize the effective Hamiltonian of the Floquet operator, we will get two eigenvalues with quasi-energy difference π. This model therefore corresponds to the π-spin-glass phase introduced in Ref. [S7].

B. Our model: the FSPT phase

Unlike Floquet time crystals introduced above, the Floquet SPT phase breaks discrete time-translation symmetry only at the boundaries. To be specific, our model of the Floquet SPT phase exhibits subharmonic response at frequency $2\pi/2T$ only at the edges but not in the bulk of the system. Here T is the period of the Floquet driving. We will now present additional theoretical analysis of our model.

1. Localized and SPT quantum states

Our FSPT phase has two distinct governing Hamiltonians during different time intervals as shown in the main text. In the first time interval, this governing Hamiltonian H_1 is the sum of one-body Pauli operators on different sites. In the second time interval, the governing Hamiltonian H_2 includes interaction among neighboring sites, which introduces the subtle many-body properties in this system.

Let us begin by studying the static Hamiltonian H_2 [S8],

$$H_2 = -\sum_k \left[J_k \hat{\sigma}_{k-1}^z \hat{\sigma}_k^x \hat{\sigma}_{k+1}^z + V_k \hat{\sigma}_k^x \hat{\sigma}_{k+1}^x + h_k \hat{\sigma}_k^x\right], \quad \text{(S5)}$$

where the parameters are chosen as in the main text. This Hamiltonian has a $\mathbb{Z}_2 \times \mathbb{Z}_2$ symmetry, corresponding to $\hat{\sigma}_k^{z,y} \longrightarrow -\hat{\sigma}_k^{z,y}$ independently on even- or odd-numbered sites, i.e. $[H_2, \prod_k \hat{\sigma}_{2k}^x] = 0$ and $[H_2, \prod_k \hat{\sigma}_{2k+1}^x] = 0$. All three-body terms $S_k = \hat{\sigma}_{k-1}^z \hat{\sigma}_k^x \hat{\sigma}_{k+1}^z$ in H_2 commute with each other, i.e. $[S_k, S_l] = 0$, and are called stabilizers.

In the extreme case $V_k = h_k = 0$, the eigenstates of this Hamiltonian are the mutual eigenstates of all stabilizers and are called cluster states. They are SPT states with $\mathbb{Z}_2 \times \mathbb{Z}_2$ symmetry. The SPT phase manifests itself in the open-boundary case: there is one effective free spin at each end of the chain. The topological nature of the eigenstates is encapsulated by the string-order parameter:

$$O_{\rm st}(l,j) = \langle \hat{\sigma}_l^z \hat{\sigma}_{l+1}^y (\prod_{k=l+2}^{j-2} \hat{\sigma}_k^x) \hat{\sigma}_{j-1}^y \hat{\sigma}_j^z \rangle, \quad \text{(S6)}$$

which takes random values $O_{\rm st}(l,j) = \pm 1$ for different eigenstates and different disorder realizations. Thus, we can define a non-local analogue of the Edwards-Anderson glass-order parameter to characterize the FSPT phase: $O_{\rm sg} = [\![O^2(l,j)]\!]$, where $[\![\,]\!]$ denotes an average over sites, states, and random realizations. The entanglement spectra of the eigenstates are degenerate. This degeneracy can serve as another manifestation of the topological nature of the phase. Furthermore, in this limit, all energy levels are exactly four-fold degenerate. The corresponding degenerate eigenstates can be divided into four groups: $\{|A_k\rangle = |\uparrow ... \uparrow\rangle\}$, $\{|B_k\rangle = |\downarrow ... \uparrow\rangle\}$, $\{|C_k\rangle = |\uparrow ... \downarrow\rangle\}$, $\{|D_k\rangle = |\downarrow ... \downarrow\rangle\}$. Here we are working in the $\hat{\sigma}_z$ basis, and the two arrows represent the effective boundary spins and the ... denotes the bulk spins. These states are related by $\prod \hat{\sigma}_{\rm odd}^x |A_k\rangle = |B_k\rangle$, $\prod \hat{\sigma}_{\rm even}^x |A_k\rangle = |C_k\rangle$, $\prod \hat{\sigma}_{\rm all}^x |A_k\rangle = |D_k\rangle$, where $\prod \hat{\sigma}_{\rm even}^x \equiv \prod_k \hat{\sigma}_{2k}^x$, $\prod \hat{\sigma}_{\rm odd}^x \equiv \prod_k \hat{\sigma}_{2k+1}^x$, and $\prod \hat{\sigma}_{\rm all}^x \equiv \prod_k \hat{\sigma}_k^x$.

When $V_k,\ h_k \neq 0$, the one- and two-body terms make the eigenstates of this Hamiltonian depart from cluster states. However, if we keep the Hamiltonian deep in the topological phase (the phase we are interested in), i.e. $J_k \gg V_k, h_k$, we can also interpret this model from a many-body localized (MBL) perspective. Unlike the $V_k = h_k = 0$ Hamiltonian with strictly localized stabilizers as the integrals of motion, in the MBL phase, the system posses a set of mutually commuting quasi-local integrals of motion. Similarly, for open boundary conditions, there exists a quasi-local effective free spin at each edge, which contains bulk components decaying exponentially with the distance from the edge. In this case, the string-order parameter and the degeneracy of the entanglement spectra can still manifest the topological nature of the eigenstates. Moreover, while the energy spectrum is no longer exactly four-fold degenerate in a finite system, it is still nearly four-fold degenerate, and the corresponding eigenstates can still be divided into the four groups introduced above.

2. The emergence of the FSPT phase

Having reviewed the properties of the static Hamiltonian H_2, let us now consider the Floquet case, wherein we periodically drive the above SPT Hamiltonian as discussed in the main text:

$$H(t) = \begin{cases} H_1, & 0 \le t < T' \\ H_2, & T' \le t < T \end{cases}, \quad \text{(S7)}$$

$$H_1 \equiv (\frac{\pi}{2} - \delta) \sum_k \hat{\sigma}_k^x, \quad \text{(S8)}$$

$$H_2 \equiv -\sum_k [J_k \hat{\sigma}_{k-1}^z \hat{\sigma}_k^x \hat{\sigma}_{k+1}^z + V_k \hat{\sigma}_k^x \hat{\sigma}_{k+1}^x + h_k \hat{\sigma}_k^x] \quad \text{(S9)}$$

where $T = 2T' = 2$.

Let us begin with the perfect case, where $\delta = 0$ and $V_k = h_k = 0$. The energy spectrum of H_2 is then perfectly four-fold degenerate. The eigenstates can be divided into four groups, i.e. $\{|A_k\rangle = |\uparrow ... \uparrow\rangle\}$, $\{|B_k\rangle = |\downarrow ... \uparrow\rangle\}$, $\{|C_k\rangle = |\uparrow ... \downarrow\rangle\}$,

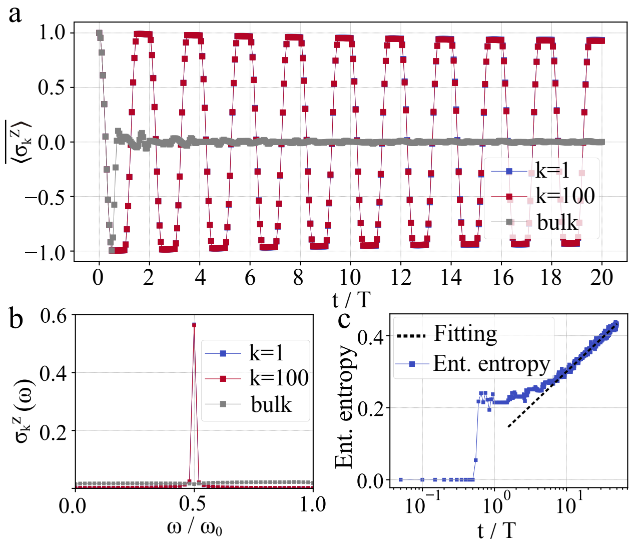

FIG. S1. The evolution of the FSPT phase for system size $L = 100$, computed using the time-evolving block decimation methods. Results shown here are averaged over 1000 random realizations, with parameters $J = 1$, $\Delta_J = 4$, $h = \Delta_h = V = \Delta_V = \delta = 0.05$. **a**. Time evolution of disorder-averaged local observables. Here, the expectation values of $\hat{\sigma}_k^z$ are multiplied by the signs of those of the initial states. For the edge spins, it is clear that $\overline{\langle\sigma_1^z\rangle}$ and $\overline{\langle\sigma_{100}^z\rangle}$ (which are right on top of each other, so that $k = 1$ is not visible) display persistent oscillations with $2T$ periodicity, manifesting the breaking of discrete time-translation symmetry. In stark contrast, bulk spin (averaged over all bulk sites) decays rapidly to zero and no symmetry breaking is observed. This is the defining feature of the FSPT phase: time-translation symmetry only breaks at the boundary, not in the bulk. **b**. Fourier spectra of $\langle\sigma_k\rangle$. We find that $\sigma_1(\omega)$ and $\sigma_{100}(\omega)$ (which are on top of each other, so that $k = 1$ is not visible) have a peak at $\omega/\omega_0 = 1/2$, where $\omega_0 = 2\pi/T$ is the driving frequency. This peak is robust and rigidly locked to $\omega_0/2$, a manifestation of the robustness of the FSPT phase. For bulk spins, there is no such peak, consistent with no symmetry breaking in the bulk. **c**. Logarithmic entanglement entropy (abbreviated as "Ent. entropy") growth. In the FSPT phase, the system is many-body localized. We thus expect logarithmic entanglement growth, which is shown in this figure. A linear fit of the entanglement entropy after $t/T = 10$ as a function of $\log t$ is plotted as a dashed line: Ent. entropy $= 0.0824 \log t + 0.1104$, with the residual sum of squares equal to 0.0215 (accurate to 4 decimal places). The entanglement has an initial quick rise until time $t \sim 1/J$. This initial rise corresponds to the expansion of wave packets to a size on the order of the localization length.

$\{|D_k\rangle = |\downarrow \dots \downarrow\rangle\}$ with $E_{A_k} = E_{B_k} = E_{C_k} = E_{D_k}$. There exists a local effective free spin at each boundary. Since the effect of $U_1 = e^{-i\pi/2\sum_k \hat{\sigma}_k^x}$ is to perfectly flip the spins at all sites, we obtain the following properties of the Floquet operator $U_F = \exp(-iH_2)\exp(-i\pi/2\sum_k \hat{\sigma}_k^x)$:

$$U_F\,|A_k\rangle = \exp(-iH_2)\,|D_k\rangle = \exp(-iE_{D_k})\,|D_k\rangle\,, \quad \text{(S10)}$$

$$U_F\,|B_k\rangle = \exp(-iH_2)\,|C_k\rangle = \exp(-iE_{C_k})\,|C_k\rangle\,, \quad \text{(S11)}$$

$$U_F\,|C_k\rangle = \exp(-iH_2)\,|B_k\rangle = \exp(-iE_{B_k})\,|B_k\rangle\,, \quad \text{(S12)}$$

$$U_F\,|D_k\rangle = \exp(-iH_2)\,|A_k\rangle = \exp(-iE_{A_k})\,|A_k\rangle\,. \quad \text{(S13)}$$

From this, we see that U_F mixes the states $|A_k\rangle$ and $|D_k\rangle$ and mixes the states $|B_k\rangle$ and $|C_k\rangle$. Within the subspace of $|A_k\rangle$ and $|D_k\rangle$, U_F has matrix form

$$\begin{bmatrix} 0 & \exp(-iE_{D_k}) \\ \exp(-iE_{A_k}) & 0 \end{bmatrix}. \quad \text{(S14)}$$

Within the subspace of $|B_k\rangle$ and $|C_k\rangle$, U_F has matrix form

$$\begin{bmatrix} 0 & \exp(-iE_{C_k}) \\ \exp(-iE_{B_k}) & 0 \end{bmatrix}. \quad \text{(S15)}$$

Therefore, in the subspace formed by $|A_k\rangle$, $|B_k\rangle$, $|C_k\rangle$, and $|D_k\rangle$, U_F has eigenvalues $\pm\exp[-i(E_{A_k} + E_{D_k})/2]$ and $\pm\exp[-i(E_{B_k}+E_{C_k})/2]$. Thus, the Floquet effective Hamiltonian has eigen-energies $(E_{A_k} + E_{D_k})/2$, $(E_{B_k} + E_{C_k})/2$, $(E_{A_k} + E_{D_k})/2 + \pi$, $(E_{B_k} + E_{C_k})/2 + \pi \mod 2\pi$. As the energy spectrum of H_2 is four-fold degenerate ($E_{A_k} = E_{B_k} = E_{C_k} = E_{D_k}$), the Floquet eigen-energies satisfy $(E_{A_k} + E_{D_k})/2 = (E_{B_k} + E_{C_k})/2$, $(E_{A_k} + E_{D_k})/2 + \pi = (E_{B_k} + E_{C_k})/2 + \pi$. Therefore, the original four-fold degeneracy breaks into two-fold degeneracy in the presence of the drive. This two-fold degeneracy is a remnant of the original topological order. As for the Floquet eigenstates, they are cat-like linear combinations of topological eigenstates: $|A_k\rangle \pm |D_k\rangle$ and $|B_k\rangle \pm |C_k\rangle$. The mutual information between the two boundary spins is $2\log 2$, indicating that there are long-range correlations between the boundaries.

When we turn on the two-body terms and the one-body terms in H_2, but still keep the system deep in the topological phase ($J_k \gg h_k, V_k$), H_2 has four nearly degenerate eigenstates related by the symmetry operations. The effective free spin at each boundary becomes quasi-local. Under Floquet driving, the near-four-fold degeneracy breaks into near-two-fold degeneracy: $(E_{A_k} + E_{D_k})/2 \approx (E_{B_k} + E_{C_k})/2$, $(E_{A_k} + E_{D_k})/2+\pi \approx (E_{B_k} + E_{C_k})/2+\pi$. Similarly, the eigenstates of the Floquet unitary are still cat-like states, and thus time-translation symmetry breaking can occur in this case. The stability of the FSPT phase will be discussed in more detail in Sec. I B 4.

3. *Dynamical properties of the FSPT phase*

Next, we will consider the evolution of this system and explicitly demonstrate the behavior of the FSPT phase.

Let us start from a product state $|\psi_0\rangle = |\downarrow \dots \uparrow\rangle$. Here ... denotes a product state of bulk spins. Because the sate of the boundary spins corresponds to the group $\{|B_k\rangle\}$, we can expand the initial state as $|\psi_0\rangle = \sum_k b_k\,|B_k\rangle$. Under the time

evolution U_F for one driving period, we have

$$\begin{aligned} U_F \left|\psi_0\right\rangle &= \exp(-iH_2)\exp(-i\pi/2\sum_j \hat{\sigma}_j^x)\sum_k b_k \left|B_k\right\rangle \\ &= \exp(-iH_2)\sum_k b_k \left|C_k\right\rangle \\ &= \sum_k b_k \exp(-iE_{C_k})\left|C_k\right\rangle , \end{aligned} \tag{S16}$$

where $|B_k\rangle = |\downarrow ... \uparrow\rangle$, $|C_k\rangle = |\uparrow ... \downarrow\rangle$. So, if we measure the edge spins in the initial state, we have $\langle\psi_0|\, \hat{\sigma}_1^z \,|\psi_0\rangle = -1$, $\langle\psi_0|\, \hat{\sigma}_N^z \,|\psi_0\rangle = 1$. After one Floquet period, the state becomes $|\psi_1\rangle = \sum_k b_k \exp(-iE_{C_k})\,|C_k\rangle$. Because $|C_k\rangle$ has definite boundary spin expectation values, we will get $\langle\psi_1|\, \hat{\sigma}_1^z \,|\psi_1\rangle = 1$, $\langle\psi_1|\, \hat{\sigma}_N^z \,|\psi_1\rangle = -1$. Similarly, after two Floquet periods, the state becomes $|\psi_2\rangle = \sum_k b_k \exp(-iE_{C_k} - iE_{B_k})\,|B_k\rangle$, and $\langle\psi_2|\, \hat{\sigma}_1^z \,|\psi_2\rangle = -1$, $\langle\psi_2|\, \hat{\sigma}_N^z \,|\psi_2\rangle = 1$. Thus, we see the the edge spins exhibit breaking of the time-translation symmetry.

As for bulk spins, assume that one bulk spin $\hat{\sigma}_j^z$ has the following expectation value in the initial product state: $\langle\psi_0|\, \hat{\sigma}_j^z \,|\psi_0\rangle = 1$. Writing $|\psi_0\rangle$ in the $|B_k\rangle$ basis, we have

$$\langle\psi_0|\, \hat{\sigma}_j^z \,|\psi_0\rangle = \sum_{k,k'} b_k b_{k'}^* \,\langle B_{k'}|\, \hat{\sigma}_j^z \,|B_k\rangle = 1. \tag{S17}$$

Since the spins of $|C_k\rangle$ are opposite to the spins of $|B_k\rangle$ at all sites, we immediately have that

$$\sum_{k,k'} b_k b_{k'}^* \,\langle C_{k'}|\, \hat{\sigma}_j^z \,|C_k\rangle = -1. \tag{S18}$$

However, the expectation value of $\hat{\sigma}_j^z$ in state $|\psi_1\rangle$ can be expressed as

$$\langle\psi_1|\, \hat{\sigma}_j^z \,|\psi_1\rangle = \sum_{k,k'} b_k b_{k'}^* \exp(-iE_{C_k} + iE_{C_{k'}})\,\langle C_{k'}|\, \hat{\sigma}_j^z \,|C_k\rangle \,. \tag{S19}$$

Comparing the last two equations, we see that, because of the extra phase factor $\exp\left(-iE_{C_k} + iE_{C_{k'}}\right)$ before each component, the $\hat{\sigma}_j^z$ will not have definite value after the Floquet time evolution and will decay to zero quickly after random averaging. Thus, bulk spins do not exhibit breaking of the time-translation symmetry.

The above derivations tell that, for our model, the edge spins exhibit discrete time-translation symmetry breaking, while bulk spins relax very fast. Thus, the time-translation symmetry breaking only occurs at the boundaries as showing in Fig. S1**a**. We stress the importance of topology here. It protects the edge spins, ensuring the robustness of the edge spins against local perturbations that respect the underlying symmetry.

Deep in the FSPT phase, the system represented by the static many-body-localized Hamiltonian H_2 has a complete set of quasi-local integrals of motion [S9]. Therefore, spins far away from each other can build significant entanglement only after exponentially long evolution time [S10]. Thus, under the Floquet time evolution, the entanglement entropy of our system exhibits logarithmic growth, as shown in Fig. S1**c**, and will eventually saturate to a value proportional to the system size.

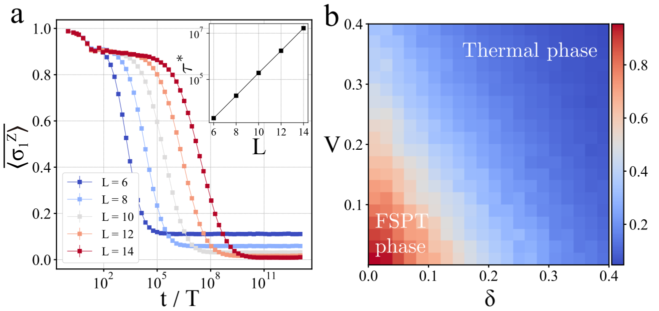

FIG. S2. The decay of boundary-spin magnetization and the phase diagram of the system. **a.** The decay of the first-spin magnetization, averaged over random disorder realizations. Here, the number of disorder realizations ranges from 3×10^4 ($L = 6$) to 10^3 ($L = 14$). The omitted parameters are chosen as in Fig. S1. We see an initial quick decay of $\overline{\langle\sigma_1^z\rangle}$, followed by a plateau that extends up to a time diverging exponentially with system size. The inset shows the exponential scaling of τ^* with system size, where τ^* is the time when the edge spin decays to $1/2$. **b.** The phase diagram of the system as a function of the parameter δ in the definition of H_1 and the average strength V of the two-body interactions. Here, we adapt the string order parameter $O_{\rm sg}$ (averaged over 100 random realizations) as the indicator. It shows that when the imperfections are not very large, the string order parameter is approximately equal to one, indicating the topological phase. The other parameters are chosen as $J = \Delta_J = 1$, $h = \Delta_h = 0.01$.

Furthermore, when system size is finite, even deep in the topological phase, the Floquet time evolution will eventually lead to the decay of the spin signal at the boundaries. Indeed, the quasi-local effective free spins at the boundaries have tails that decay exponentially into the bulk. When system size is finite, these tails have an exponentially small overlap, which leads to the relaxation of the two effective free spins, with the lifetime diverging exponentially with system size. We demonstrate this phenomenon numerically in Fig. S2**a**.

We note that the subharmonic response of the edge spins cannot be observed by examining $\langle\sigma_1^x(t)\rangle$ or $\langle\sigma_L^x(t)\rangle$ with initial states prepared as product states along the x direction. In this case, $\overline{\langle\sigma_1^x(t)\rangle}$ and $\overline{\langle\sigma_L^x(t)\rangle}$ will decay to zero quickly and show no persistent oscillation. This can be understood by considering H_2 in the idealized cluster-state limit ($V_k = h_k = 0$). Taking $\langle\sigma_1^x(t)\rangle$ as an example, the edge operators for the decoupled left edge mode (which behaves as a spin-1/2) read: $\Sigma_1^x = \sigma_1^x\sigma_2^z$, $\Sigma_1^y = \sigma_1^y\sigma_2^z$, and $\Sigma_1^z = \sigma_1^z$. The x component of the left edge mode in fact involves σ_2^z, which will result in the decay of $\overline{\langle\sigma_1^x\rangle}$. An alternative way to understand this is by noting the fact that σ_1^x does not commute with H_2, which is different from the case of $\langle\sigma_1^z\rangle$, where σ_1^z indeed commutes with H_2 in the cluster-state limit.

4. The stability of the FSPT phase

The above considerations rely on the fact that, during each period, we perfectly flip spins at all sites. To show that the FSPT phase is indeed stable, we should make sure its defining properties hold even for an imperfect drive. We follow arguments similar to those introduced in Ref. [S5].

We showed above that, in the perfect-drive case ($\delta = 0$), the eigenstates of the Floquet evolution operator are cat-like states $\left|\psi_{\pm}^{AD}\right\rangle = |A_k\rangle \pm |D_k\rangle$ and $\left|\psi_{\pm}^{BC}\right\rangle = |B_k\rangle \pm |C_k\rangle$. We say that an effective short-range correlated topological state satisfies $\langle\psi|\,\hat{\sigma}_1\hat{\sigma}_N\,|\psi\rangle - \langle\psi|\,\hat{\sigma}_1\,|\psi\rangle\,\langle\psi|\,\hat{\sigma}_N\,|\psi\rangle \to 0$. Obviously, $|A_k\rangle\,, |B_k\rangle\,, |C_k\rangle\,, |D_k\rangle$ are short-range correlated topological states, but the Floquet eigenstates $\left|\psi_{\pm}^{AD}\right\rangle$ and $\left|\psi_{\pm}^{BC}\right\rangle$ are all long-range correlated, with different quasienergies. Then, any experimentally prepared short-range correlated state (such as a product state) can only be formed by taking a superposition of those long-range-correlated Floquet eigenstates with different quasienergies. Thus, after one period of Floquet evolution, local observables at the edge will not be invariant, signaling a breaking of discrete time-translation symmetry.

Now we add local $\mathbb{Z}_2 \times \mathbb{Z}_2$-symmetric perturbations into the system, such as an imperfect drive ($\delta \neq 0$), two-body interactions ($V_k \neq 0$), and single-body terms ($h_k \neq 0$). As long as the system is in an MBL phase, a local perturbation will significantly affect only nearby sites. Thus, we expect that the long-range correlations in the eigenstates of the Floquet unitary will not disappear. In fact, there exists a quasi-local $\mathbb{Z}_2 \times \mathbb{Z}_2$-symmetric unitary operator U, which constructs the perturbed Floquet eigenstates from the unperturbed Floquet eigenstates. Since U is quasi-local and symmetric, it cannot destroy the long-range boundary correlations of the unperturbed Floquet eigenstates. (Note, however, that perturbations that break the protecting symmetry but maintain MBL can destroy the FSPT phase, as discussed in Ref. [S11].) Therefore, time-translation-symmetry breaking can also occur in the locally perturbed system. To explicitly show that the FSPT phase is indeed a phase, we use the string order parameter $O_{\rm sg}$ as the indicator to plot in Fig. S2**b** the phase diagram with respect to the drive imperfection δ and the average strength V of two-body interactions.

We mention that with large V and ΔV, there will be a trivial MBL phase. It is also worthwhile to study the transition between the trivial MBL phase and the thermal phase, and the transition between the FSPT phase and the trivial MBL phase. In the main text, we use the variance of the subharmonic spectral peak height to locate the transition point between the FSPT phase and the thermal phase. This method cannot be used to distinguish the trivial MBL phase and the thermal phase, since for both phases there is no breaking of discrete time translational symmetry and hence no subharmonic spectral peak. One may use other quantities, such as level statistics and entanglement entropy, to probe the transition between the trivial MBL phase and the thermal phase, similar to the static scenarios without periodic driving [S12]. To distinguish the FSPT phase and the trivial MBL phase, one can also exploit the above mentioned Edwards-Anderson-like string order parameter [S13]. In this paper, we focus on distinguishing the FSPT phase and the thermal phase, while leaving the study of other possible transitions for future investigation.

5. Mapping to free fermions when $V_k = 0$

In this section, we review the mapping of the time-periodic Hamiltonian $H(t)$ defined in Eq. (1) in the main text [equivalently Eq. (S7)] to free fermions when the two-body interactions V_k are set to zero. This is achieved by a Jordan-Wigner transformation whereby a spin operator on site k is represented in terms of two Majorana operators, $\hat{\alpha}_k$ and $\hat{\beta}_k$. The Majorana operators are defined via the nonlocal mapping

$$\hat{\alpha}_k = \left(\prod_{j<k}\hat{\sigma}_j^x\right)\hat{\sigma}_k^z, \qquad \hat{\beta}_k = i\,\hat{\alpha}_k\hat{\sigma}_k^x. \tag{S20}$$

Under this transformation, we have

$$\hat{\sigma}_k^x = -i\hat{\alpha}_k\hat{\beta}_k \tag{S21a}$$

and

$$\hat{\sigma}_{k-1}^z\hat{\sigma}_k^x\hat{\sigma}_{k+1}^z = -i\hat{\beta}_{k-1}\hat{\alpha}_{k+1}. \tag{S21b}$$

The mapping thus results in redefined Hamiltonians

$$H_1 = -i(\frac{\pi}{2} - \delta)\sum_k \hat{\alpha}_k\hat{\beta}_k \tag{S22a}$$

and

$$H_2 = i\sum_k\left(J_k\,\hat{\beta}_{k-1}\hat{\alpha}_{k+1} + h_k\,\hat{\alpha}_k\hat{\beta}_k\right). \tag{S22b}$$

Note that H_2 can be rewritten as

$$\begin{aligned} H_2 &= H_2^{\rm odd} + H_2^{\rm even} \\ &= i\sum_{k\ \rm odd}\left(J_{k+1}\,\hat{\beta}_k\hat{\alpha}_{k+2} + h_k\,\hat{\alpha}_k\hat{\beta}_k\right) \\ &\quad + i\sum_{k\ \rm even}\left(J_{k+1}\,\hat{\beta}_k\hat{\alpha}_{k+2} + h_k\,\hat{\alpha}_k\hat{\beta}_k\right), \end{aligned} \tag{S23}$$

which corresponds to two decoupled Kitaev chains [S14], one on the odd and one on the even sublattice. The $\mathbb{Z}_2 \times \mathbb{Z}_2$ symmetry of $H(t)$ then manifests itself as the separate conservation of the two fermion parity operators

$$P_{\rm odd\ (even)} = \prod_{k\ \rm odd\ (even)}(i\hat{\alpha}_k\hat{\beta}_k), \tag{S24}$$

with eigenvalues ± 1.

When $T' = 1$ and $\delta = h_k = 0$, the time-dependent Hamiltonian $H(t)$ maps onto two copies of the fixed-point model for the nontrivial class D FSPT phase studied in Ref. [S15].

To see this, note that, when $\delta = 0$ and $T' = 1$, we have (up to an unimportant overall phase factor)

$$\exp\left(-iT'H_1\right) = P_{\text{even}} P_{\text{odd}}. \tag{S25}$$

Thus, we obtain the Floquet operator (setting $T = 2T' = 2$)

$$U_F = P_{\text{even}}\, e^{-iH_2^{\text{even}}} P_{\text{odd}}\, e^{-iH_2^{\text{odd}}}. \tag{S26}$$

If we additionally set $h_k = 0$, this Floquet operator is just a product of two decoupled copies of the class D model considered in Ref. [S15]. The model studied in this work is thus expected to remain in this universality class for any small perturbations that respect the $\mathbb{Z}_2 \times \mathbb{Z}_2$ symmetry of Eq. (S26), including finite δ, h_k, and V_k.

II. DETAILS OF THE TEBD METHOD

We numerically simulate the time evolution process of the FSPT phase using the time-evolving block decimation (TEBD) method. This method was proposed for the time evolution of matrix product states (MPS) [S16, S17] and is a variant of the density matrix renormalization group (DMRG) algorithm [S18, S19]. At the heart of the TEBD method lies the Trotter-Suzuki decomposition of the time-evolution operator $U(\Delta t)$ of a short-range interacting system over a small time interval Δt. Usually, we can represent the operator $U(\Delta t)$ in the matrix-product-operator (MPO) form with small Trotter error, and then repeatedly apply it on the MPS representing the current state $|\psi(t)\rangle$ of the system to implement the time evolution.

Our FSPT phase has two distinct Hamiltonian operators in different time intervals as shown above. For the first time interval, the corresponding Hamiltonian is the sum of one-body operators on different sites. So the evolution operator is a direct product of one-body evolution operators

$$U_1(t) = e^{-itH_1} = e^{-it(\pi/2-\delta)\hat{\sigma}_1^x} \otimes \ldots \otimes e^{-it(\pi/2-\delta)\hat{\sigma}_k^x} \otimes \ldots, \tag{S27}$$

which can be represented as an MPO directly. To obtain the corresponding expectation values of local observables at different times, we also decompose the time evolution operator of an entire time interval T' into several small time intervals Δt. We show the implementation of $U_1(\Delta t)$ in Fig. S3**a**.

For the second time interval, the Hamiltonian H_2 consists of multiple short-range interaction terms: $H_2 = -\sum_k [J_k \hat{\sigma}_{k-1}^z \hat{\sigma}_k^x \hat{\sigma}_{k+1}^z + V_k \hat{\sigma}_k^x \hat{\sigma}_{k+1}^x + h_k \hat{\sigma}_k^x]$. Thus, we can approximate the time-evolution operator using Trotter-Suzuki decomposition $U_2(t) \approx [U_2(\Delta t)]^{t/\Delta t} = \left[e^{-i\Delta t H_2}\right]^{t/\Delta t}$ with $\Delta t \ll t$. To efficiently construct the MPO representation of $U_2(\Delta t)$, we group together terms in H_2 that commute with each other. The three-body operators are all stabilizer operators and commute with each other. For the two-body terms, they also commute with each other. For one-body terms, all of them are act on different sites and thus commute with each other. For simplicity, we denote $A = -\sum_k J_k \hat{\sigma}_{k-1}^z \hat{\sigma}_k^x \hat{\sigma}_{k+1}^z, B = -\sum_k V_k \hat{\sigma}_k^x \hat{\sigma}_{k+1}^x, C = -\sum_k h_k \hat{\sigma}_k^x$ and obtain

$$\begin{aligned} U_2(\Delta t) =& e^{-i\Delta t(A+B+C)} \\ =& e^{-i\Delta t C} e^{-i\Delta t(B+A)} e^{-i\Delta t^2 [C,B+A]} \\ &+ \mathcal{O}(\Delta t^3) \\ =& e^{-i\Delta t C} e^{-i\Delta t B} e^{-i\Delta t A} + \mathcal{O}(\Delta t^2). \end{aligned} \tag{S28}$$

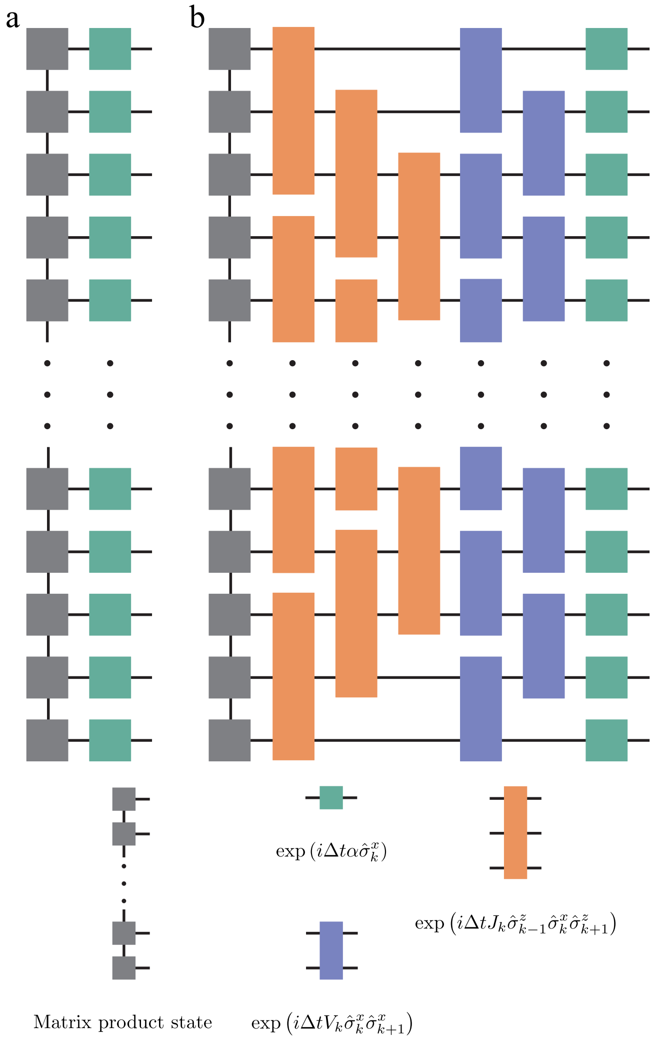

FIG. S3. Pictorial illustration of the implementation of the time-evolution unitary, where a connected wire between different blocks means contraction of indices. **a.** Implementation of $U_1(\Delta t)$. The gray blocks represent the current state in MPS form, and the green blocks represent the time evolution unitary consisting of one-body operators ($\alpha = -(\pi/2 - \delta)$). **b.** Implementation of $U_2(\Delta t)$. The gray blocks represent the current state in MPS form; the orange blocks represent the time-evolution unitary consisting of three-body operators arranged in three groups; the blue blocks represent the time-evolution unitary consisting of two-body operators arranged in two groups; and the green blocks represent the time evolution unitary consisting of one-body operators ($\alpha = h_k$). These unitaries are applied to the the current state layer by layer.

Thus, the time-evolution operator for H_2 over time interval t is approximated by

$$U(t) \approx [U_2(\Delta t)]^{t/\Delta t} = \left(e^{-i\Delta t C} e^{-i\Delta t B} e^{-i\Delta t A}\right)^{t/\Delta t} + \mathcal{O}(\Delta t). \qquad \text{(S29)}$$

Furthermore, to make the numerical simulation more efficient, the implementation of three-body terms and the two-body terms can be accomplished layer by layer, wherein each layer only contains operators with no overlapping support, so that they can be applied to the MPS in parallel. We emphasize that, since the Trotter error is of order Δt, the time interval Δt should be small enough to avoid large TEBD error. The implementation of $U_2(\Delta t)$ is showed in Fig. S3**b**.

III. EXPERIMENTAL DETAILS

A. Quantum circuit ansatz

Algorithm 1: Neuroevolution Method

Output: Quantum circuit ansatz approximating target unitary.
Input: Elementary gate set $\mathcal{S}$, evolution unitary U_{target} and threshold β.
$\mathcal{G}$ = Direct_Graph($\mathcal{S}$);
$\mathcal{C}$ = Random_Generation_of_Quantum_Circuit($\mathcal{G}$);
$\mathcal{L}$ = Optimization($\mathcal{C}$, U_{target});
while $\min\{\mathcal{L}\} > \beta$ **do**
    $\mathcal{C}$ = Quantum_Circuit_Extension($\mathcal{C}$, $\mathcal{G}$);
    $\mathcal{L}$ = Optimization($\mathcal{C}$, U_{target});
end
return $\text{argmin}_{\mathcal{C}}\{\mathcal{L}\}$;

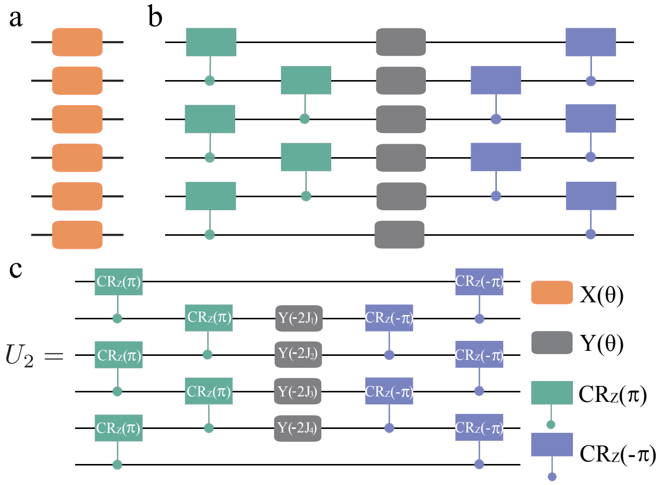

FIG. S4. Quantum circuit ansatzes used in our experiments. **a.** The circuit ansatz for the time-evolution unitary over the first time interval. **b.** The circuit ansatz for the time-evolution unitary over the second time interval, where the system is deep in the topological phase. **c.** The circuit ansatz for the time-evolution unitary over the second time interval in the cluster limit ($V_k = h_k = 0$), where the system contains only three-body interactions: $U_2 = \exp\left(i\sum_k J_k \hat{\sigma}^z_{k-1}\hat{\sigma}^x_k\hat{\sigma}^z_{k+1}\right)$.

Algorithm 2: Optimization for a quantum circuit

Output: Optimal parameters of the given quantum circuit.
Input: A quantum circuit C, evolution unitary U_{target} and learning rate γ.
Randomly initialize $\boldsymbol{\theta}$;
$U_{\text{circuit}}(\boldsymbol{\theta})$ = Unitary(C, $\boldsymbol{\theta}$);
$L = 1 - \text{Tr}\left[U^{\dagger}_{\text{target}} U_{\text{circuit}}(\boldsymbol{\theta})\right]/d$;
while $L > 0.001$ **do**
    $\boldsymbol{\theta} = \boldsymbol{\theta} - \gamma\nabla_{\boldsymbol{\theta}} L$;
    $U_{\text{circuit}}(\boldsymbol{\theta})$ = Unitary(C, $\boldsymbol{\theta}$);
    $L = 1 - \text{Tr}\left[U^{\dagger}_{\text{target}} U_{\text{circuit}}(\boldsymbol{\theta})\right]/d$;
end
return $\boldsymbol{\theta}$;

To observe the FSPT phase on a digital superconducting quantum computer, we need to decompose the time-evolution unitary into a quantum circuit consisting of a series of experimentally implementable quantum gates. Due to the direct-product structure of the evolution unitary $U_1(t) = e^{-itH_1}$ in the first time interval, this unitary can be represented as a quantum circuit using a layer of rotation gates along the x axis. Thus, it can be constructed and implemented relatively easily. As for the second time interval, the interaction among different sites takes the time-evolution unitary far away from a direct product form, making things a little different.

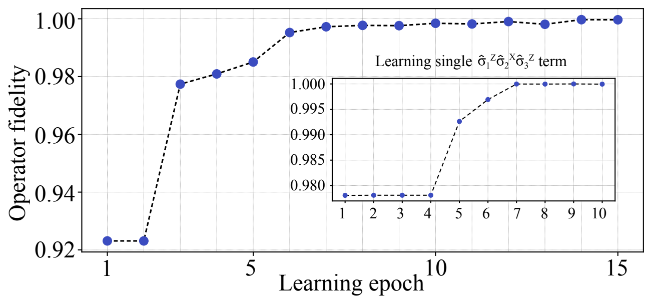

FIG. S5. Circuit ansatz learning procedure. This figure shows the learning procedure of approximating a six-qubit evolution unitary. The indicator is the operator fidelity $\mathrm{Tr}\left[U_{\mathrm{target}}^{\dagger}U_{\mathrm{circuit}}\left(\boldsymbol{\theta}\right)\right]/d$. The final fidelity is larger than 0.999. The inset shows the learning procedure of approximating a single $\hat{\sigma}_1^z\hat{\sigma}_2^x\hat{\sigma}_3^z$ term, where the final fidelity is larger than 0.9999.

With the progress of research on variational quantum circuits, we are able to adapt this method to construct the quantum circuit of the second-time-interval unitary. Variational quantum circuits are a powerful tool that has been intensively investigated in recent years. Algorithms based on variational quantum circuits hold great potential in the noisy intermediate-scale quantum era. There are many algorithms based on variational parameterized quantum circuits, such as the variational quantum eigen-solver [S20], the quantum neural network [S21], etc.. The major distinction between standard quantum circuits and variational quantum circuits is that the gates composing a variational quantum circuit are not fixed. They can be modified by tuning their parameters using different parameter-updating algorithms. As these parameters are updated, the unitary implemented by the variational circuit is also updated. We terminate the updating procedure when a satisfactory result is obtained.

Our target is to find a variational quantum circuit, with some fine-tuned parameters, that approximates to high precision the evolution unitary $U_2(t) = e^{-itH_2}$ in the second time interval. We accomplish this target in two steps: find an implementable variational quantum circuit ansatz that can be used to represent the target unitary and keep updating the parameters contained in this circuit ansatz to find a good approximation of the desired unitary.

We use the neuroevolution method [S22] to find a suitable variational circuit architecture. The elementary gates used in our experiments are variable-angle single-qubit rotation gates, $X(\theta)$, $Y(\theta)$, $Z(\theta)$ (θ is the variational parameter), and a variable-angle control-rotation gate along the z axis $\mathrm{CR}_z(\theta)$ (θ is the variational parameter). Each of these gates contains a variational parameter, the rotation angle. These gates can form various quantum circuit layers. i.e. quantum circuits with depth equal to one. Using the method of Ref. [S22], we construct out of these layers a directed graph, so that a quantum circuit can be represented as a path in this graph. To find the desired circuit, we follow the following procedure: 1) Randomly generate several variational quantum circuits of fixed depth based on the directed graph; 2) Update parameters contained in those quantum circuits using a gradient-based algorithm to minimize the loss function $L(\boldsymbol{\theta}) = 1 - \mathrm{Tr}\left[U_{\mathrm{target}}^{\dagger}U_{\mathrm{circuit}}(\boldsymbol{\theta})\right]/d$, where U_{target} is the evolution unitary over the second time interval $U_2(\Delta t)$, $U_{\mathrm{circuit}}(\boldsymbol{\theta})$ is the unitary represented by the current quantum circuit with variational parameters $\boldsymbol{\theta}$, and d is the dimension of the corresponding Hilbert space; 3) Chose quantum circuits with small values of the loss function and extend them based on the directed graph to generate new circuits; 4) Iterate processes 2) and 3) until the loss function is below a desired threshold. The circuit ansatz giving the smallest value of the loss function is regarded as the optimal ansatz representing the evolution unitary and is adapted in our experiments. We show the pseudo-code of this algorithm in Algorithm 1.

In the evolution procedure, we will repeatedly optimize the current circuits to approximate the target unitary. This optimization is also required when we apply the found circuit ansatz to approximate the evolution unitary of a particular disorder realization of H_2. For a target unitary U_{target} and a particular circuit ansatz, we begin with this ansatz containing randomly generated variational parameters $\boldsymbol{\theta}$. Then, the gradient of the loss function $L(\boldsymbol{\theta})$ with respect to those variational parameters is computed and is used to update the current parameters $\boldsymbol{\theta}^{(n+1)} = \boldsymbol{\theta}^{(n)} - \gamma\nabla_{\boldsymbol{\theta}^{(n)}}L$, where γ is a given learning rate (we usually chose $0.001 \leq \gamma \leq 0.01$). In our calculation, we iterate this optimization procedure until the operator fidelity [S23] satisfies $\mathrm{Tr}\left[U_{\mathrm{target}}^{\dagger}U_{\mathrm{circuit}}\left(\boldsymbol{\theta}\right)\right]/d \geq 0.999$ ($L(\boldsymbol{\theta}) \leq 0.001$). We then take the quantum circuit with the final parameters as the approximation of the target unitary. We show the pseudo-code of this algorithm in Algorithm 2.

As an illustration, in Fig. S5, we show the learning procedure of a six-qubit unitary using algorithms above. The target unitary is the evolution unitary of the Hamiltonian in the second time interval: $H = -\sum_k\left[J_k\hat{\sigma}_{k-1}^z\hat{\sigma}_k^x\hat{\sigma}_{k+1}^z + V_k\hat{\sigma}_k^x\hat{\sigma}_{k+1}^x + h_k\hat{\sigma}_k^x\right]$, where J_k are uniformly chosen from $[0,2]$, V_k are uniformly chosen from $[0,0.02]$, and h_k are uniformly chosen from $[0,0.02]$. We first construct the directed graph of six qubits, where each node represents a specific layer of quantum gates and only layers that can not be merged together are connected, e.g. the layer of R_x gates is connected to the layer of R_z gates, but is not connected to itself. Then, we randomly generate five initial paths consisting of two connected nodes in this directed graph, which represent circuits consisting of two layers of quantum gates, as described in Algorithm 1. We then train those initial circuits to approximate the target unitary using Algorithm 2. Then, if the approximation results do not fulfill the predefined threshold precision, we will randomly generate subsequent paths consisting of two connected nodes for each initial path, where the first node in the subsequent path is connected in the directed graph to the last node in the current path, and extend the current paths by appending the subsequent paths. We repeat Algorithm 2 again to approximate the target unitary. The extension and optimization procedures are subsequently

carried out until at least one of the circuits can approximate the target unitary to a predefined precision. Using the same method, we can also construct the circuit representation of a single $\hat{\sigma}_1^z\hat{\sigma}_2^x\hat{\sigma}_3^z$ term. We find that the final approximation precision can be very high (usually we can set the threshold precision to 0.9999). Using a straightforward simplification (removing gates with small parameters, merging nearby quantum gates, etc.), we can construct an exact representation of this term. The learning procedure is shown in the inset of Fig. S5.

The quantum circuit ansatzes used in our experiments are shown in Fig. S4. We notice that the quantum circuit for the evolution unitary over the second time interval has a sandwich form $U_2(\Delta t) \approx WD(\boldsymbol{\theta})W^\dagger$, where $D(\boldsymbol{\theta})$ is a layer of single-qubit rotation gates with $\boldsymbol{\theta}$ being the evolution-time-dependent parameters. We note that the circuit structure in Fig. S4 **b** has an appealing merit: imagining that we first use this circuit to simulate the evolution of the system under H_2 for a small time interval Δt, then for the subsequent time interval Δt the $\mathrm{CR}_z(\pi)$ gates in the current circuit will cancel with the $\mathrm{CR}_z(-\pi)$ gates in the preceding circuit. Besides, since the middle layer of this evolution circuit consists of single-qubit rotation gates, they can merge with the following single-qubit rotation gates. As a result, for $2\Delta t$ time evolution the circuit maintains the same structure, and we only need to double the angles for the single-qubit $Y(\theta)$ rotations to simulate the evolution. Thus, for one driving period the depth of the corresponding quantum circuit can be maintained to be six. We mention that this merit carries over to the circuit structure in Fig. S4 **c** as well.

We emphasize that this optimization procedure is suitable for small systems. On the other hand, because of the exponential growth of the dimension of the Hilbert space, the optimization for large systems is impractical. It is helpful that the quantum circuit ansatz found using the neuroevolution method can exactly represent the evolution unitary $U_2(t) = e^{-itH_2}$ when H_2 has no two-body operators and no one-body operators (as shown in Fig. S4**c**). This indicates that we can analytically construct the corresponding quantum circuits for arbitrarily many qubits when $V_k = h_k = 0$, regardless of what values J_k and δ have. In fact, in this case, we can find an exact simple one-to-one mapping between J_k and the variational rotation angles in Fig. S4**c**. In our simulations and experiments, for systems of $L \leq 8$, the two-body terms and one-body terms are considered and the parameters in the corresponding quantum circuits are obtained using the above-described gradient-based optimization method. For 14-qubit systems, we only consider the stabilizer terms in H_2 and exactly construct the corresponding quantum circuits.

B. Device overview and measurement setup

To illustrate the idea of the FSPT phase, we run the experiments on two different superconducting quantum processors, referred to as version α (Fig. S8) and version β (main text Fig. 2). Both of them are flip-chip devices hosting an array of 6×6 qubits distributed in a square lattice. We select a chain of up to $L = 14$ (26) qubits in processor α (β). To realize high-fidelity controlled-Z (CZ) gates, we adopt the tunable-coupler architecture [S24] to mediated nearest-neighbor qubit-qubit interactions, i.e., individual couplers are inserted between neighboring qubits with the qubit-coupler coupling strengths designed to be around 130 MHz for qubits at 6.5 GHz. All qubits (couplers) are of transmon type, with anharmonicities around 250 (350) MHz and maximum resonance frequencies around 7 (10.5) GHz. Each qubit has its own control line, which takes microwave (XY) inputs for rotating the qubit state around the x- or y-axis and flux-bias (Z) pulses for tuning the qubit frequency and rotating the qubit state around the z-axis; each coupler is frequency tunable via its own flux bias (Z) line, which guarantees that the effective coupling strength between two neighboring qubits at 6.5 GHz can be dynamically turned on, up to -25 MHz, or off, ≤ 0.25 MHz. Each qubit capacitively couples to its own readout resonator, designed in the frequency range around 4.3 GHz, for qubit state measurement. 9 readout resonators share one readout transmission line (TL) running across the processor chip, and 4 readout TLs can cover all 36 qubits in the processor.

The processors were fabricated using the flip-chip recipe: all qubits and couplers are located on the sapphire substrate (top chip); most of the control/readout lines and readout resonators are located on the silicon substrate (bottom chip). For processor α, these two chips have lithographically defined base wirings, junction loops, and airbridges made of aluminum, and are galvanically connected via indium bumps with titanium under-bump metallization, as described elsewhere [S25]. For processor β, aluminum was replaced by tantalum for most structures except for junction loops and airbridges, and indium bumps were grown on tantalum without under-bump metallization. The indium bumps were formed by the lift-off method with 9 μm-thick indium deposited on both chips, after which these two chips were aligned and bonded together at room temperature to complete the flip-chip device. The indium bumps in our processor are not only for ground connectivity, but also for passing through control signals from the bottom chip to the top chip where the qubits are located.

The processor was loaded into a multilayer printed circuit board (PCB) enclosure, which was then mounted inside a dilution refrigerator (DR) with the base temperature down to 15 mK. Figure S6 shows the schematics of the control/readout electronics and wiring setup. In this setup, the XY microwave signals and fast Z pulses synthesized by digital-to-analog converters (DACs) are first joined together at room temperature, then attenuated and filtered at multiple cold stages of DR, and later combined with the slow Z (DC) pulses via home-made bias-tees at the mixing chamber stage of DR before being transmitted into the qubit control lines. The multiplexed readout signals are also heavily attenuated and filtered before going into the readout TLs of the processor to retrieve the qubit state information. To boost the signal-to-noise ratio

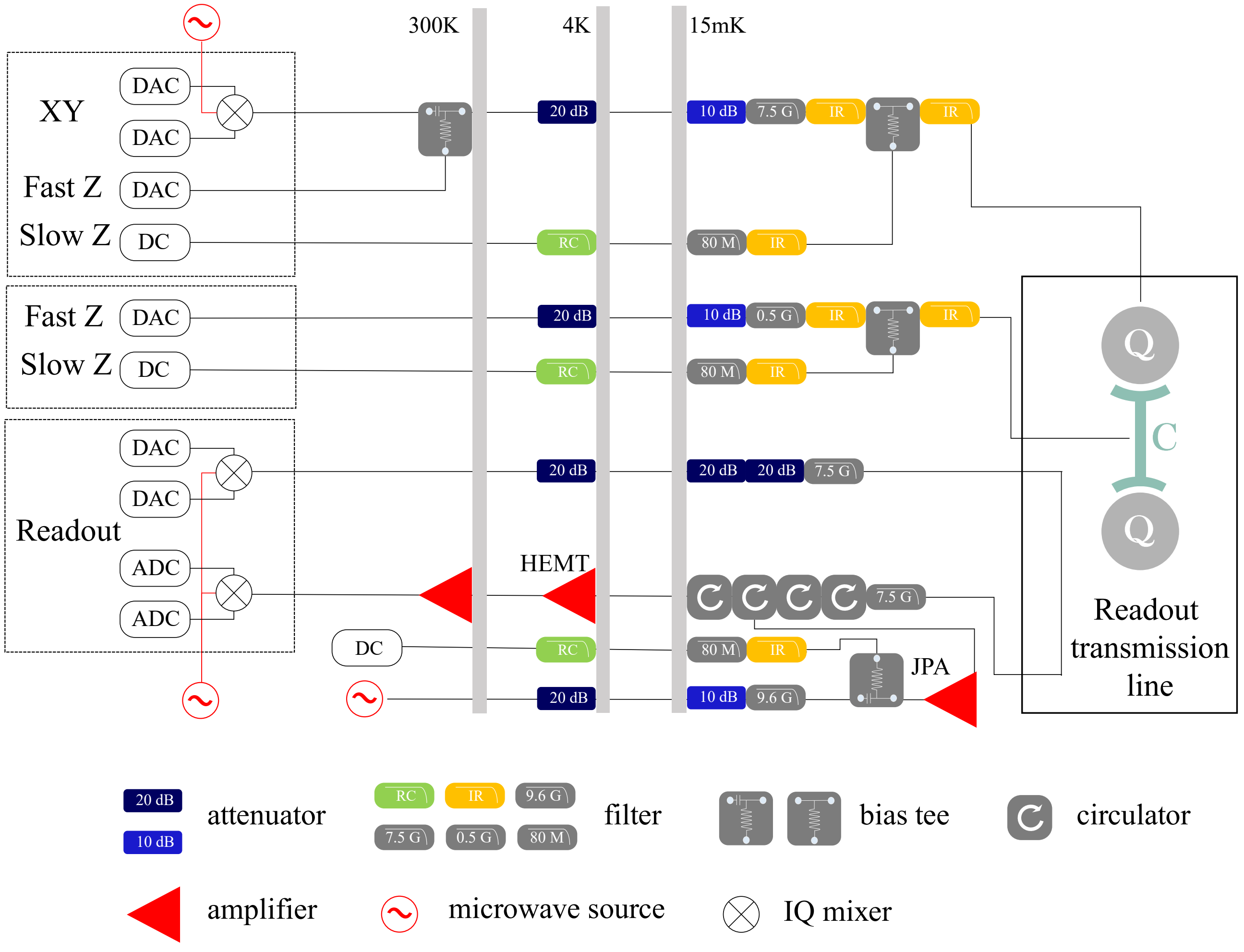

FIG. S6. Electronics and wiring setup illustrating how to synthesize and transmit the control/readout signals. Each qubit has three control channels: XY (microwave), fast Z (flux), and slow Z (flux). Each coupler has two control channels: fast Z and slow Z. Readout pulses are generated similarly to the XY signals and are passed through the processor via the readout TLs. All control and readout lines are well-attenuated and filtered for noise shielding and delicate control.

(SNR), output signals from TLs are sequentially amplified by a Josephson parametric amplifier (JPA), a high electron mobility transistor (HEMT) amplifier, and room temperature (RT) amplifiers before being demodulated by analog-to-digital converters (ADCs) with 10-bit vertical resolution and 1.0 GS/s sampling rate. An arbitrary microwave signal can be generated by mixing the DAC outputs with continuous microwave using IQ mixer. DACs used to synthesize XY microwave signals and fast Z pulses in this experiment have 14-bit vertical resolution and 300 MHz output bandwidth. Slow Z (DC) pulses are generated by commercial 16-bit DACs with maximum outputs of ± 2.5 V.

C. Single- and Two-qubit gates

Single-qubit gates used in this experiment include X(θ), Y(θ), and Z(θ), which rotate the qubit state by an arbitrary angle θ around x-, y-, and z-axis, respectively. We realize X(θ) and Y(θ) by controlling the amplitude and phase of XY microwave pulses, and implement Z(θ) via the virtual Z gate [S26]. Single-qubit gate errors are characterized by simultaneous randomized benchmarking, yielding an average gate fidelity above 0.99 for both processors (see Tab. S1 and Tab. S2). We note that in both tables, the notation is defined as follows. ω_j^0 is the maximum frequency of Q_j at zero flux bias. ω_j is the idle frequency where we initialize Q_j in $|0\rangle$ and subsequently apply single-qubit gates. η_j is Q_j's anharmonicity, which is approximately a constant within the frequency range

relevant to this experiment. $\left(\omega_j^{\mathrm{A\,(B)}}, \omega_{j+1}^{\mathrm{A\,(B)}}\right)$ is a list of two frequencies for two neighboring qubits in group A (group B), chosen such that $|11\rangle$ and $|02\rangle$ in the two-qubit subspace have nearly the same energy for a CZ gate; the CZ gates for qubit pairs in the same group A (B) are implemented simultaneously when executing the multilayer quantum circuit to simulate the FSPT phase. ω_j^{m} is the readout frequency of Q_j where we apply readout pulses to excite Q_j's readout resonator for quantum state measurement. ω_j^{r} is the resonant frequency of Q_j's readout resonator. $T_{1,j}$ and $T_{2,j}^*$ are the energy relaxation time and Ramsey dephasing time of Q_j, respectively. $F_{0,j}$ and $F_{1,j}$ are the readout fidelity values for Q_j prepared in $|0\rangle$ and $|1\rangle$, respectively; these fidelity values are used to correct raw probabilities to eliminate readout errors as done previously [S27]. We note that $F_{0,j}$ and $F_{1,j}$ are actually the averaged probability values obtained by repeating the same state preparation and measurement sequence 3000 times, and the associated statistical errors are typically around 0.0055 and 0.0095, respectively. e_{sq} lists the single-qubit gate errors obtained by simultaneous randomized benchmarking. $e_{\mathrm{CZ}}^{\mathrm{A(B)}}$ list the CZ gate errors obtained by both individual and simultaneous randomized benchmarking for qubit pairs in group A (B). We note that the qubit parameters may slowly drift over time [S28, S29].

The basic structure to implement the CZ gate consists of two flux tunable qubits and one flux tunable coupler, which are, respectively, denoted as Q_1, Q_2, and C here for clarity of description. The effective coupling strength is composed of a direct coupling strength between two qubits and a part mediated by the coupler, which can be continuously adjusted by controlling the flux or frequency of the coupler. The Hamiltonian of this three body system is written as

$$H/\hbar = \sum_{i=1,2,c} \omega_i a_i^\dagger a_i + \frac{\eta_i}{2} a_i^\dagger a_i^\dagger a_i a_i + \sum_{i<j} g_{ij} \left(a_i - a_i^\dagger\right)\left(a_j - a_j^\dagger\right), \quad \text{(S30)}$$

where $a_i^\dagger$ and a_i are raising and lowering operators, and g_{ij} is the coupling strength between each pair in $\{Q_1, Q_2, C\}$. The effective coupling strength between qubits is

$$\tilde{g} = g_{1c} g_{2c} \left(\frac{\omega_c}{\omega_1^2 - \omega_c^2} + \frac{\omega_c}{\omega_2^2 - \omega_c^2}\right) + g_{12}. \quad \text{(S31)}$$

In Fig. S7**a**, we plot the dynamic range of $\tilde{g}$ (bottom panel) processed using the two-qubit swap dynamics after initializing Q_1-Q_2 in $|10\rangle$, which shows that the effective coupling strength is tunable in the range from -25 MHz to ≤ 0.25 MHz. Experimentally, we can apply single-qubit gates while tuning the frequency of the coupler to around 10.5 GHz to turn off $\tilde{g}$.

To realize the CZ gate, we apply a flux bias (fast Z) pulse to steer the coupler's frequency along the following trajectory: 10.5→7.3→10.5 GHz. Meanwhile, we turn on the fast Z pulses to bring a pair of qubits from their idle frequencies to the pair of values $\left(\omega_j^{\mathrm{A(B)}}, \omega_{j+1}^{\mathrm{A(B)}}\right)$ (see Tab. S1 for processor β and Tab. S2 for processor α), chosen such that $|11\rangle$ and $|02\rangle$ in the two-qubit subspace have nearly the same energy. After a finite period for this diabatic interaction, a unitary two-qubit gate equivalent to a CZ gate up to trivial single-qubit phase factors can be obtained as

$$\begin{pmatrix} 1 & 0 & 0 & 0 \\ 0 & e^{i\phi_1} & 0 & 0 \\ 0 & 0 & e^{i\phi_2} & 0 \\ 0 & 0 & 0 & e^{i\phi_3} \end{pmatrix}. \quad \text{(S32)}$$

A sine-decorated square pulse with the amplitude $A = z_0 \times \left[1 - r + r\sin\left(\pi \frac{t}{t_{\mathrm{gate}}}\right)\right]$ is used for the coupler in order to minimize state leakage. Experimentally, we fix $r = 0.3$ and only fine-tune the parameter z_0. All pulses are digitally smoothed by convolving them via a Gaussian window with $\sigma = 2$ ns before applying our pulse calibration routines [S30]. The CZ gate pulse duration is 30 ns, and there are additional 5 ns padding times before and after the 30-ns gate in compensation for the finite small tails of the smoothed pulse.

Individual CZ gates are calibrated following the procedure below:

1. Optimize coupler Z bias amplitude for minimum state leakage: We initialize Q_1-Q_2 in $|11\rangle$ and fix their frequency detuning at $\omega_1 - \omega_2 \approx -2\pi \times 250$ MHz, following which we apply the sine-decorated square pulse with a total length of 40 ns to the coupler. We search for the optimized pulse amplitude z_0 which maximizes the $|1\rangle$-state population for Q_1, i.e., minimum state leakage. In Fig. S7**b**, we plot the whole landscape of state leakage as functions of the Z bias amplitudes of both the coupler and Q_1, where the black solid line indicates how we sweep the coupler Z pulse amplitude.

2. Optimize phase factors: We fix the coupler Z pulse and sweep Q_1's Z pulse amplitude using different initial states to calculate the three phase factors in Eq. (S32), aiming at the condition $\phi_3 - \phi_2 - \phi_1 = \pi$. The black dashed line in Fig. S7**b** shows the routine of how we sweep the qubit Z pulse amplitude. We apply virtual Z gates to remove the trivial single-qubit phases.

3. Fine-tune gate parameters according to randomized benchmarking: We choose the randomized benchmarking sequence fidelity as a goal function to optimize relevant gate parameters, including the Z pulse amplitudes of both qubits and the coupler, and the single-qubit phases. We use the Nelder-Mead method to speed up the parameter optimization process.

TABLE S1. Device parameters for processor β with 26 qubits.

Qubit	Q_1	Q_2	Q_3	Q_4	Q_5	Q_6	Q_7	Q_8	Q_9	Q_{10}	Q_{11}	Q_{12}	Q_{13}	Q_{14}
$\omega_j^0/2\pi$ (GHz)	6.717	6.780	6.882	6.635	6.820	6.914	6.816	6.613	6.771	6.832	6.689	6.836	6.768	6.668
$\omega_j/2\pi$ (GHz)	6.450	6.650	6.850	6.540	6.508	6.630	6.790	6.580	6.676	6.800	6.450	6.650	6.708	6.540
$\eta_j/2\pi$ (GHz)	0.244	0.250	0.245	0.246	0.249	0.248	0.251	0.242	0.239	0.247	0.244	0.242	0.242	0.245
$\left(\omega_j^{\text{A}}, \omega_{j+1}^{\text{A}}\right)/2\pi$ (GHz)	6.439, 6.678		6.835 , 6.601		6.412 , 6.647		6.809 , 6.559		6.552 , 6.784		6.438 , 6.670		6.756 , 6.531	
$\left(\omega_j^{\text{B}}, \omega_{j+1}^{\text{B}}\right)/2\pi$ (GHz)		6.627 , 6.858		6.488 , 6.251		6.563 , 6.806		6.482 , 6.721		6.741 , 6.511		6.520 , 6.750		
$\omega_j^{\text{m}}/2\pi$ (GHz)	6.466	6.273	6.600	6.170	6.520	6.640	6.815	6.580	6.676	6.750	6.452	6.610	6.670	6.540
$\omega_j^{\text{r}}/2\pi$ (GHz)	4.433	4.266	4.496	4.364	4.413	4.314	4.335	4.440	4.297	4.500	4.385	4.514	4.522	4.317
$T_{1,j}$ (μs)	34	23	21	29	26	34	29	29	30	7	34	29	28	33
$T_{2,j}$ (μs)	2.2	1.7	2.7	4.1	2.5	1.4	2.5	4.4	3.0	2.2	2.1	2.0	3.2	3.0
$F_{0,j}$	0.973	0.917	0.947	0.952	0.968	0.939	0.892	0.922	0.923	0.941	0.956	0.955	0.951	0.947
$F_{1,j}$	0.930	0.872	0.910	0.907	0.887	0.904	0.878	0.850	0.860	0.871	0.919	0.898	0.861	0.935
e_{sq} (%)	0.33	0.55	1.28	0.44	0.77	0.69	1.32	0.38	1.07	1.37	0.85	0.36	0.55	0.34
e_{CZ}^{A} (%) (Indiv.)	0.94		1.84		1.68		3.12		2.12		1.49		1.23	
e_{CZ}^{B} (%) (Indiv.)		1.56		2.39		5.13		2.70		2.17		2.62		
e_{CZ}^{A} (%) (Simu.)	1.28		1.98		1.86		2.94		2.20		1.30		0.91	
e_{CZ}^{B} (%) (Simu.)		1.08		1.08		4.46		2.44		2.49		1.86		

Qubit	Q_{14}	Q_{15}	Q_{16}	Q_{17}	Q_{18}	Q_{19}	Q_{20}	Q_{21}	Q_{22}	Q_{23}	Q_{24}	Q_{25}	Q_{26}
$\omega_j^0/2\pi$ (GHz)		6.893	6.744	6.853	6.771	6.727	6.941	6.897	6.738	6.722	6.737	6.732	6.784
$\omega_j/2\pi$ (GHz)		6.605	6.650	6.708	6.430	6.330	6.630	6.600	6.540	6.482	6.650	6.508	6.630
$\eta_j/2\pi$ (GHz)		0.249	0.246	0.253	0.260	0.246	0.241	0.248	0.250	0.250	0.242	0.246	0.243
$\left(\omega_j^{\text{A}}, \omega_{j+1}^{\text{A}}\right)/2\pi$ (GHz)		6.460 , 6.697		6.760 , 6.525		6.379 , 6.602		6.752 , 6.515		6.429 , 6.662		6.471 , 6.699	
$\left(\omega_j^{\text{B}}, \omega_{j+1}^{\text{B}}\right)/2\pi$ (GHz)	6.386 , 6.618		6.522 , 6.761		6.467 , 6.231		6.700 , 6.478		6.571 , 6.335		6.627 , 6.403		
$\omega_j^{\text{m}}/2\pi$ (GHz)		6.575	6.650	6.695	6.430	6.115	6.634	6.600	6.470	6.120	6.656	6.502	6.680
$\omega_j^{\text{r}}/2\pi$ (GHz)		4.430	4.357	4.568	4.280	4.454	4.376	4.493	4.520	4.410	4.427	4.375	4.414
$T_{1,j}$ (μs)		33	37	27	41	33	40	34	41	38	34	28	37
$T_{2,j}$ (μs)		2.4	4.7	3.3	2.7	2.7	2.9	1.6	3.9	2.1	4.3	2.9	2.4
$F_{0,j}$		0.929	0.946	0.952	0.937	0.977	0.953	0.961	0.969	0.981	0.957	0.959	0.963
$F_{1,j}$		0.908	0.894	0.909	0.880	0.926	0.911	0.905	0.900	0.890	0.908	0.919	0.895
e_{sq} (%)		0.67	0.36	0.46	0.43	0.44	0.55	0.57	0.55	0.59	0.38	0.70	0.40
e_{CZ}^{A} (%) (Indiv.)		1.69		1.14		2.72		3.48		1.33		1.71	
e_{CZ}^{B} (%) (Indiv.)	1.67		1.74		1.81		3.38		1.21		1.43		
e_{CZ}^{A} (%) (Simu.)		1.57		1.09		2.05		2.51		1.18		1.22	
e_{CZ}^{B} (%) (Simu.)	1.49		1.18		2.02		3.74		1.31		0.97		

TABLE S2. Device parameters for processor α with 14 qubits.

Qubit	Q_1	Q_2	Q_3	Q_4	Q_5	Q_6	Q_7	Q_8	Q_9	Q_{10}	Q_{11}	Q_{12}	Q_{13}	Q_{14}
$\omega_j^0/2\pi$ (GHz)	7.021	6.970	7.000	6.864	6.840	7.028	6.819	6.879	6.770	6.854	6.818	6.962	6.925	6.970
$\omega_j/2\pi$ (GHz)	6.450	6.730	6.890	6.651	6.565	6.750	6.676	6.600	6.520	6.620	6.721	6.893	6.838	6.960
$\eta_j/2\pi$ (GHz)	0.230	0.248	0.248	0.242	0.255	0.239	0.288	0.247	0.251	0.246	0.241	0.250	0.247	0.252
$\left(\omega_j^{\mathrm{A}}, \omega_{j+1}^{\mathrm{A}}\right)/2\pi$ (GHz)	6.414, 6.656		6.893, 6.651		6.275, 6.516		6.632, 6.868		6.349, 6.585		6.717, 6.957		6.684, 6.920	
$\left(\omega_j^{\mathrm{B}}, \omega_{j+1}^{\mathrm{B}}\right)/2\pi$ (GHz)		6.667, 6.898		6.651, 6.412		6.894, 6.657		6.766, 6.528		6.485, 6.722		6.910, 6.676		
$\omega_j^{\mathrm{m}}/2\pi$ (GHz)	6.110	6.198	5.608	6.651	5.552	6.309	6.722	5.997	5.812	5.828	6.323	5.736	6.181	6.423
$\omega_j^{\mathrm{r}}/2\pi$ (GHz)	4.357	4.194	4.119	4.200	4.097	4.343	4.323	4.223	4.262	4.206	4.152	4.269	4.182	4.402
$T_{1,j}$ (μs)	25	22	28	36	11	27	27	30	22	33	25	37	13	29
$T_{2,j}^*$ (μs)	1.0	1.7	4.5	2.5	3.8	2.2	1.2	1.6	0.8	2.1	3.1	2.8	5.8	14.0
$F_{0,j}$	0.950	0.955	0.945	0.888	0.951	0.951	0.961	0.956	0.868	0.880	0.959	0.935	0.980	0.970
$F_{1,j}$	0.876	0.862	0.834	0.888	0.886	0.942	0.859	0.900	0.890	0.905	0.900	0.898	0.919	0.937
e_{sq} (%)	0.49	0.45	1.26	0.72	0.38	0.69	0.66	0.47	0.84	0.60	0.33	0.55	0.55	0.45
$e_{\mathrm{CZ}}^{\mathrm{A}}$ (%) (Indiv.)	1.06		0.22		1.79		0.74		0.99		0.37		1.09	
$e_{\mathrm{CZ}}^{\mathrm{B}}$ (%) (Indiv.)		0.29		1.24		0.59		1.77		0.78		1.68		
$e_{\mathrm{CZ}}^{\mathrm{A}}$ (%) (Simu.)	3.46		0.99		3.00		0.76		2.03		0.79		1.33	
$e_{\mathrm{CZ}}^{\mathrm{B}}$ (%) (Simu.)		0.76		0.51		0.81		2.29		0.97		2.05		

D. Scalability of the digital simulation approach

In the main text, we have plotted the dynamics of the disorder-averaged local magnetization obtained from the quantum processor β in Fig. 2, with 26 qubits. As mentioned above, we have also run the experiment on the processor α with 14 qubits, and the result is plotted in Fig. S8. Here, we measure $\overline{\langle \sigma_j^z \rangle}$ at different time steps within a period, so as to see in more detail how this quantity evolves. This will increase the experimental efforts and we thus only evolve the system up to 20 cycles. From Fig. S8, it is clear that the edge spins oscillate with a stable subharmonic response, whereas the bulk magnetization decays quickly to zero. Comparing the results shown in Fig. 2 in the main text with those in Fig. S8, we find that they both demonstrate the theoretically predicted behavior to the same level of accuracy, despite the fact that the system size for Fig. 2 is almost twice as that for Fig. S8. This shows the scalability of our digital simulation approach.

IV. NUMERICAL SIMULATIONS CONSIDERING EXPERIMENTAL IMPERFECTIONS

Experimentally realized single- and two-qubit gates suffer from experimental imperfections such as decoherence, pulse distortions, and cross-talk effects, which need to be considered in our numerical simulations. As numerically taking into account all experimental subtleties for a faithful comparison with the experimental data is extremely hard and time consuming, we choose a simplified model of our experimental imperfections that nevertheless captures our experimental data reasonably well. In particular, when simulating the dynamics of the FSPT and EDSPT phases, we apply a small amount of random over/under rotations on the single-qubit gate matrices, which introduces an average coherent error of r=0.005 that corresponds to the measured single-qubit gate fidelity on average.

Experimental two-qubit gates have an average gate error above 0.01. Since both qubit energy relaxation and pure dephasing times are typically more than 10 μs, which are significantly longer than the typical gate durations, the error contribution due to decoherence is small (less than 0.003) [S31], and for simplicity we numerically ignore its effect. We estimate that state leakage outside of the qubit computational space of $\{|0\rangle, |1\rangle\}$ is a major source of errors, since several factors can contribute to state leakage errors, such as the short-timescale and long-timescale pulse distortions in flux lines, the level shifting due to dephasing of the flux sensitive qubits, the cross-talk interaction between non-neighboring qubits, and the $|2\rangle$ state interference [S32]. Here we numerically obtain a quantum process χ matrix by slightly shifting the optimal interacting frequencies of the qubits and the coupler, which

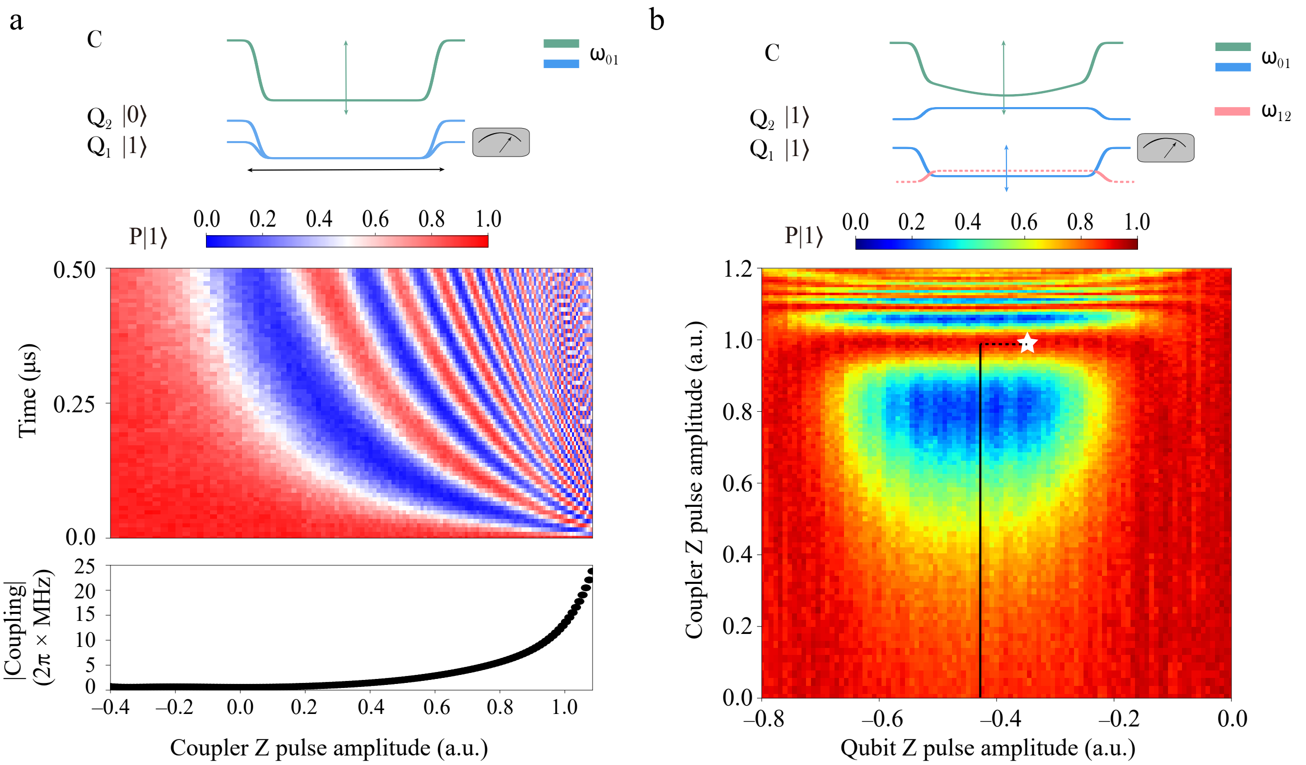

FIG. S7. Two-qubit CZ gate. **a**, two-qubit swap dynamics showing the dynamical range of the effective coupling strength as tuned by the coupler. **b**, $|1\rangle$-state population landscape for Q_1 resulting from the $|11\rangle$ and $|02\rangle$ interaction after initializing Q_1-Q_2 in $|11\rangle$. The white star marks the vicinity of the gate parameters used for the CZ gate, and lines indicate how we sweep Z parameters to approach this vicinity.

results in the state leakage error with a magnitude that corresponds to the experimentally measured infidelity. The χ matrix has a fidelity of 0.987, which is used throughout our numerical simulations. We emphasize that our numerical approach is not a faithful description of our experiments, but it reproduces the experimental data reasonably well.

V. QUANTUM STATE TOMOGRAPHY

We use quantum state tomography (QST) to reconstruct the reduced density matrix ρ_{half}, which takes $3^{N/2}$ operations for an N-qubit system. More specifically, for $N = 10$, we first generate the SPT state and then evolve the system under $H(t)$ for time $t = nT$ $(n = 0, 1, \ldots)$. Then we apply 243 combinations of the tomographic operations $[I, X/2 = X(\pi/2), Y/2 = Y(\pi/2)]^{\otimes 5}$ on one half of the chain, either $\{Q_1, Q_2, Q_3, Q_4$ and $Q_5\}$ in ρ_{A} or $\{Q_6, Q_7, Q_8, Q_9$ and $Q_{10}\}$ in ρ_{B}, before measuring these 5 qubits. We repeat each tomographic operation and readout 90000 times to obtain the $2^5 = 32$ raw binary probabilities for all 5 qubits, which are corrected to eliminate readout errors [S27]. ρ_{half} can be reconstructed via the least-squares optimization. Note that, with our measurement setup, this procedure becomes notably time-consuming as N increases above 5. Figure S9 shows the entanglement spectra of the initial SPT state for open and periodic boundary conditions. The sharp contrast between the two- and four-fold degeneracies for open and periodic boundary conditions, respectively, highlights the topological nature of the SPT state. A similar comparison between the two- and four-fold degeneracies after one driving period can be found in the main text Fig. 3**b**.

VI. DYNAMICS OF ENTANGLEMENT

Unlike thermal phases without disorder or Anderson localized phases without interaction, where entanglement grows ballistically [S33–S35] or saturates to an area law at long times, respectively, the entanglement entropy of an MBL system grows logarithmically and saturates to a volume law in the long-time limit [S10]. In our experiment, we also extract the entanglement dynamics, through a full quantum state tomography of the reduced density matrix describing one half of the system. In Fig. S10**a**, we plot the reduced density matrix ρ_{half} for a single random instance of the Hamiltonian at the end of one driving period. Using the tomographically extracted ρ_{half} at different times, we extract the desired information about entanglement growth for the FSPT phase. Our results are plotted in Fig. S10**b**. From this figure, it is clear

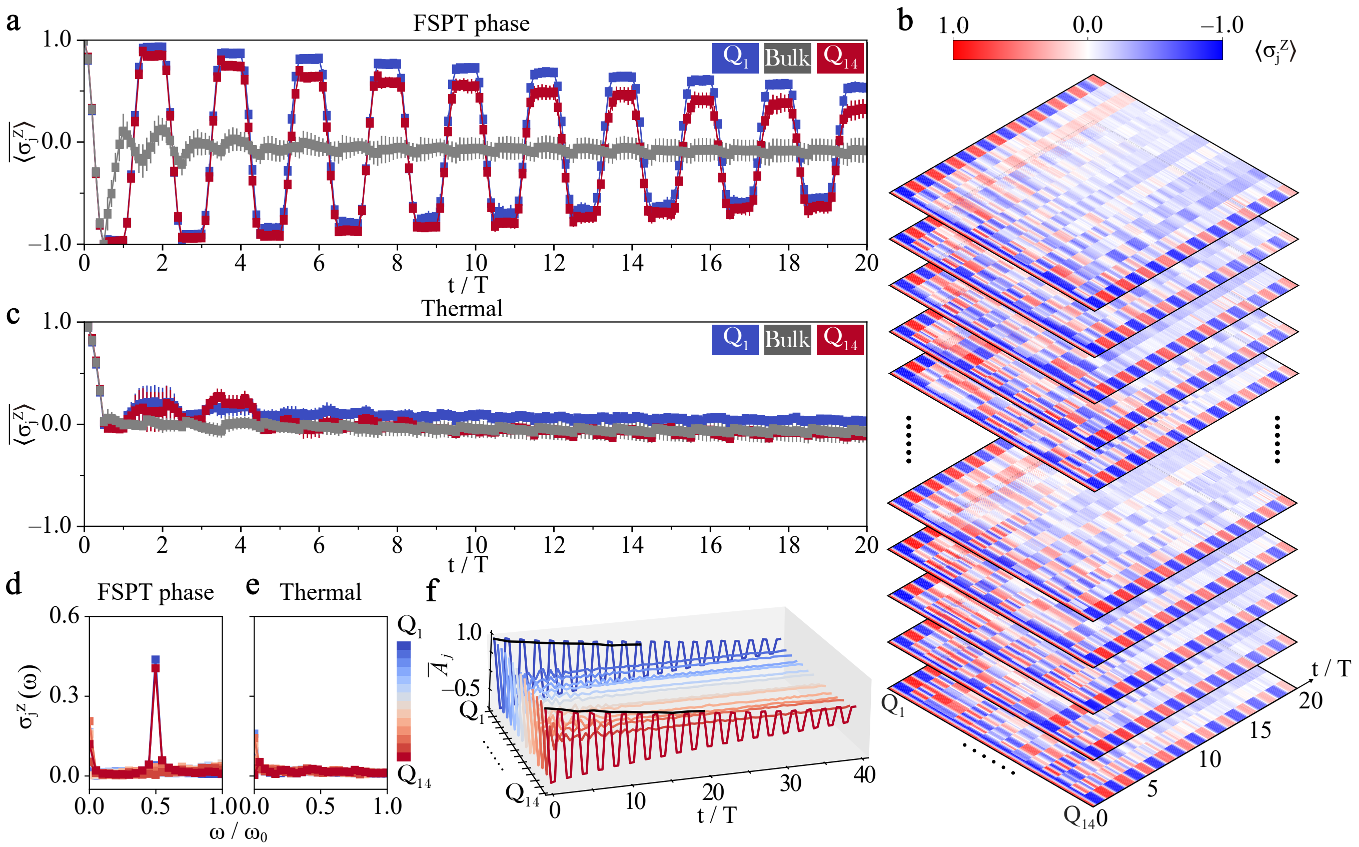

FIG. S8. **Observation of an FSPT phase in the processor α with $L = 14$ qubits.** **a**, Time evolution of disorder-averaged local magnetizations under the Hamiltonian $H(t)$ [see Eq. (1) in the main text] with $J = \Delta_J = 1$, $V = h = \Delta_V = \Delta_h = 0$, and $\delta = 0.01$, and the initial state $|0\rangle^{\otimes L}$. The data shown is averaged over 20 random disorder instances, and the error bars represent the standard error of the mean over disorder samples. **b**, The dynamics of local magnetizations for different random instances, with each layer corresponding to a specific random instance. **c**, Magnetization dynamics deep in the thermal phase ($J = \Delta_J = 1$, $V = h = \Delta_V = \Delta_h = 0$, and $\delta = 0.8$). **d**, Fourier transform of experimentally measured $\overline{\langle\sigma_j^z(t)\rangle}$ shown in **a**. **e**, Fourier spectra of $\overline{\langle\sigma_j^z(t)\rangle}$ shown in **c**. **f**, Time-dependence of the autocorrelator $\overline{A}_j = \overline{\langle\Psi_0|\sigma_j^z(t)\sigma_j^z(0)|\Psi_0\rangle}$ for up to 40 cycles, obtained from averaging over 20 random instances deep in the FSPT phase. Here, the initial states are random product states in the computational basis. The black solid lines show the results of "echo" circuits for the two boundary qubits.

that, in the thermal phase, entanglement grows quickly and saturates to a maximal volume law ($\sim \frac{L}{2}\ln 2$). In contrast, in the FSPT phase, entanglement grows much slower. Numerical simulations with two-qubit gate errors agree well with the experimental results. However we are not able to observe the logarithmic growth of entanglement both because the system size is too small and because the gate fidelities are not high enough. We display the numerical simulation for the ideal case (solid lines in Fig. S10**b**), which grows much slower than the experimental results. Observation of logarithmic growth demands not only a significant improvement of gate fidelities and a substantial increase of the coherence time, but also a more efficient and scalable approach to measure entanglement for a many-body system.

VII. EXTENDED MODELS

To exhibit the general applicability of our methods, we also implement other dynamical SPT phases in our devices using similar strategies.

A. FSPT phase of a driven Ising chain

The first extended model is the driven Ising chain possessing a $\mathbb{Z}_2$ Ising symmetry as described in Refs. [S7, S36]. We implement in our devices an FSPT phase of this periodically driven Ising chain with a time-periodic Hamitonian defined as follows:

$$H(t) = \begin{cases} H_{\text{single}} = \sum_k g_k \hat{\sigma}_k^x, & 0 < t \le T', \\ H_{\text{Ising}} = \sum_k J_k \hat{\sigma}_k^z \hat{\sigma}_{k+1}^z, & T' < t \le T, \end{cases} \tag{S33}$$

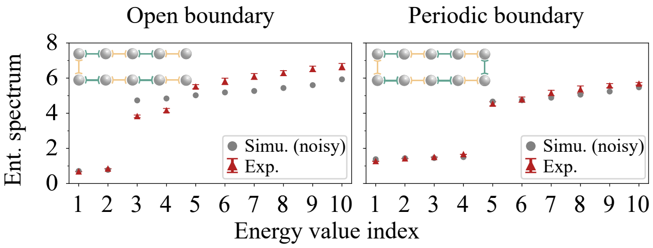

FIG. S9. Entanglement (abbreviated as “Ent.”) spectra of a random 10-qubit SPT state prepared in our experiment, with both open and periodic boundary conditions as sketched in the insets. The triangles with error bars (red) are experimental entanglement energies. The dots (grey) are simulation results with CZ error and readout noise included. For each boundary condition, only one random SPT state is generated. A total of 90000 single-shot measurements are used to reconstruct ρ_{half} and calculate the ent. spectra error bars. The two- and four-fold degeneracy (in the case of open and periodic boundary conditions, respectively) of the low-lying entanglement levels is a characteristic feature of the topological nature of these states. Note that, for a 10-qubit chain, there are a total of 32 energy values. Here we sort these values in ascending order and only display the lowest 10 of them as labelled by their energy value indices.

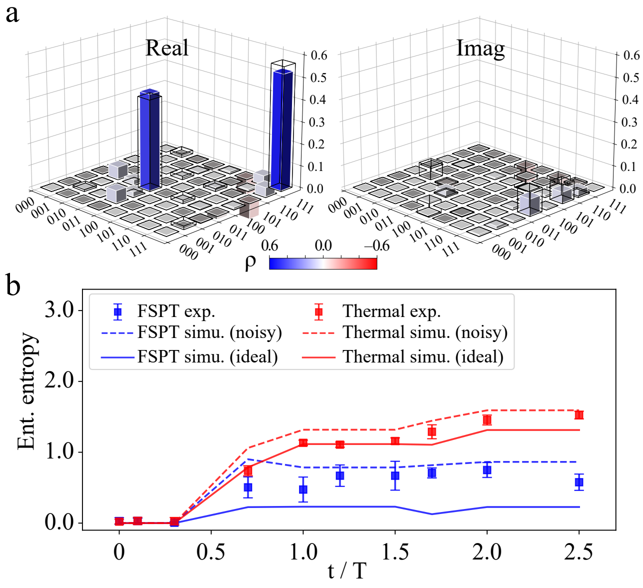

FIG. S10. Entanglement dynamics. **a**, Tomography of the reduced density matrix for the second half of a six-qubit chain after one driving period of one disorder realization. The real and imaginary parts are shown in the left and right panel, respectively. **b**, Entanglement (abbreviated as “Ent.”) entropy growth in both the thermal phase (red squares with 1σ error bars, $\delta = 0.65$, $J = 1$, $\Delta_J = 0.05$ and $V = \Delta_V = h = \Delta_h = 0.01$) and the FSPT phase (blue squares with 1σ error bars, $\delta = 0.01$, $J = \Delta_J = 1$ and $V = \Delta_V = h = \Delta_h = 0.01$), averaged over 9 disorder realizations. The dashed (solid) lines are numerical simulations with (without) CZ error included.

Here, we set $T = 2T' = 2$, and choose parameters g_k, J_k according to the target phase. As studied in Ref. [S7], this model supports four dynamical phases determined by the choice of parameters: the paramagnet (PM) phase, the 0-spin-glass (0SG) phase, the π-spin-glass (πSG) phase, and the 0π-paramagnet (0πPM) phase. We carry out the experiments on a one-dimensional open chain, and set $J_k = \frac{\pi}{2} - 0.1$ and uniformly choose g_k from $[\frac{\pi}{4} - \frac{\pi}{3}, \frac{\pi}{4} + \frac{\pi}{3}]$. This parameter region corresponds to the 0πPM phase, which is also an FSPT phase.

Using the above parameters, the evolution unitary in the first time interval $[0, T']$ is a rotation about the x-axis for each spin, and the evolution unitary in the second time interval $(T', T]$ for an open chain with length L can be rewritten as:

$$U_{\text{Ising}} = e^{-iH_{\text{Ising}}} \approx \hat{\sigma}_1^z \left(\prod_{k=2}^{k=L-1} \hat{\sigma}_k^z \hat{\sigma}_k^z \right) \hat{\sigma}_L^z = \hat{\sigma}_1^z \hat{\sigma}_L^z. \quad \text{(S34)}$$

Thus, the Floquet unitary is represented as $U_F \approx \hat{\sigma}_1^z \hat{\sigma}_L^z U_{\text{single}} = \hat{\sigma}_1^z \hat{\sigma}_L^z e^{-i \sum_k g_k \hat{\sigma}_k^x}$. For an initial product state in the x basis: 1) for the bulk sites, the Floquet evolution unitary will not change their states except adding extra global phases since $[\hat{\sigma}_k^x, U_F] \approx 0$ for $k = 2, 3, ..., L-1$; 2) for the edge sites 1 and L, the Floquet evolution unitary will flip the states in the x basis since $\{\hat{\sigma}_k^x, U_F\} \approx 0$ for $k = 1, L$. So, the eigenstates of the whole system under this time-periodic Hamiltonian are cat-like states with odd/even superpositions of x spins at the boundaries. This Floquet phase also exhibits time translational symmetry breaking at the boundaries. This property can be intuitively understood: starting from product states in the x basis, after one evolution period, the edge states are flipped, while the bulk states are unchanged. So, the edge spins return to their original states after two periods. This gives the $2T$ periodicity of the edge states. From the Heisenberg picture, this property can be exhibited explicitly: $\hat{\sigma}_k^x(nT) = (U_F^\dagger)^n \hat{\sigma}_k^x (U_F)^n \approx \hat{\sigma}_k^x$, for $k = 2, 3, ..., L-1$, and $\hat{\sigma}_k^x(nT) = (U_F^\dagger)^n \hat{\sigma}_k^x (U_F)^n \approx (-1)^n \hat{\sigma}_k^x$, for $k = 1, L$.

The 0πPM phase is a novel FSPT phase. With open boundary conditions, this phase supports no bulk long-range order, but maintains coherent edge states. In the fermion picture, the eigenstates can be described as two Majorana modes at each edge: one at quasienergy 0 and the other at quasienergy π [S7]. Furthermore, in the equilibrium situation, one requires at least a $\mathbb{Z}_2 \times \mathbb{Z}_2$ symmetry to realize a static SPT phase. In this driven Ising model, the governing Hamiltonian has a $\mathbb{Z}_2$ symmetry but has no $\mathbb{Z}_2 \times \mathbb{Z}_2$ symmetry. The Floquet drive introduces another $\mathbb{Z}$ component coming from the discrete time-transnational symmetry. The $\mathbb{Z}_2 \times \mathbb{Z}$ symmetry of the Floquet unitary shows that the 0πPM phase is an intrinsically non-equilibrium FSPT phase [S37–S39].

In our experiments, we implement 12 random realizations of this model, and observe the dynamics of the spin chain evolved under the Hamiltonian described above, with initial states being random product states in the x basis. We measure the local magnetization $\hat{\sigma}_k^x$ at different evolution times and show the results in Extended Data Fig. 5.

B. Emergent dynamical SPT phase of a quasiperiodically driven chain

The second extended model we implement in our devices has no microscopic symmetry. It is a quasiperiodically driven AKLT (Affleck-Kennedy-Lieb-Tasaki)-like chain within an emergent dynamical SPT (EDSPT) phase. We adapt the theoretical analysis from Ref. [S40] and the experimental implementation from Ref. [S41]. The Hamiltonian of this model is defined on a spin chain of length L:

$$H_x = \frac{J}{2}\sum_{k=1}^{L/2}\hat{\sigma}^x_{2k-1}\hat{\sigma}^x_{2k} + \frac{1}{2}\sum_{k=1}^{(L-2)/2} K^x_k\hat{\sigma}^x_{2k}\hat{\sigma}^x_{2k+1} + \frac{1}{2}\sum_{k=1}^{L}\boldsymbol{B}^x_k\cdot\hat{\boldsymbol{\sigma}}_{\boldsymbol{k}}, \tag{S35}$$

$$H_z = \frac{J}{2}\sum_{k=1}^{L/2}\hat{\sigma}^z_{2k-1}\hat{\sigma}^z_{2k} + \frac{1}{2}\sum_{k=1}^{(L-2)/2} K^z_k\hat{\sigma}^z_{2k}\hat{\sigma}^z_{2k+1} + \frac{1}{2}\sum_{k=1}^{L}\boldsymbol{B}^z_k\cdot\hat{\boldsymbol{\sigma}}_{\boldsymbol{k}}, \tag{S36}$$

where $\hat{\boldsymbol{\sigma}}_{\boldsymbol{k}} = [\hat{\sigma}^x_k, \hat{\sigma}^y_k, \hat{\sigma}^z_k]$, J is the pulse parameter, K^x_k and K^z_k are coupling parameters uniformly chosen from $[0, 4\pi]$, and $\boldsymbol{B}^x_k$ and $\boldsymbol{B}^z_k$ are random fields whose norms are chosen uniformly from $[0, 0.3]$ and whose random directions are also chosen uniformly. The corresponding evolution unitaries implemented using quantum circuits are recursively defined as

$$U^{(1)} = U_x = e^{-iH_x},\ U^{(2)} = U_xU_z = e^{-iH_x}e^{-iH_z}, \tag{S37}$$

$$U^{(\nu)} = U^{(\nu-2)}U^{(\nu-1)}, \tag{S38}$$

where $U^{(\nu)}$ represents the evolution unitary at Fibonacci time $t_\nu = F_\nu$, with F_ν being the ν-th element of the Fibonacci sequence.

At the solvable point $J = \pi$ and $\boldsymbol{B}^x_k = \boldsymbol{B}^z_k = 0$, the edge evolution of this quasiperiodic drive is a sequence of spin flips:... $(\hat{\sigma}^x\hat{\sigma}^z\hat{\sigma}^x\hat{\sigma}^x\hat{\sigma}^z)\,(\hat{\sigma}^x\hat{\sigma}^x\hat{\sigma}^z)\,(\hat{\sigma}^x\hat{\sigma}^z)\,(\hat{\sigma}^x)$. So, the edge states in the x (z) basis will be flipped by z (x) pulses respectively, leading to a three-fold periodicity of the edge spins when the system is measured at Fibonacci times. This non-trivial edge dynamics is stable to generic, local, and quasiperiodic-in-time perturbations. When one chooses J near π but not exactly equal to π, and $\boldsymbol{B}^x_k$, $\boldsymbol{B}^z_k \neq 0$, the evolution unitary U_N acquires a locally dressed $\mathbb{Z}_2 \times \mathbb{Z}_2$ symmetry [S40, S41] at least in the short-time regime, despite the fact that the random fields can break all microscopic symmetries in this model. The details of the locally dressed symmetry are determined by the parameters chosen for H_x and H_z, indicating it is a dynamical emergent symmetry. During the evolution, this emergent symmetry will protect a pair of coherent edge modes for a long time, even when the non-topologically-protected bulk modes have fully decohered. This can be observed if one measures the edge spins at Fibonacci times, as described above in the solvable limit.

In our experiments, we prepare the initial states by randomly choosing product states in the x (z) basis. Then, we evolve the states and measure the local magnetization $\hat{\sigma}^x_k$ ($\hat{\sigma}^z_k$) at Fibonacci times. We implement 12 random realizations of this model, and evolve 10 random initial states in the x (z) basis for each random realization in our experiments. The measurement results are shown in Extended Data Fig. 6.

[S1] A. Shapere and F. Wilczek, Phys. Rev. Lett. **109**, 160402 (2012).
[S2] F. Wilczek, Phys. Rev. Lett. **109**, 160401 (2012).
[S3] P. Bruno, Phys. Rev. Lett. **111**, 070402 (2013).
[S4] H. Watanabe and M. Oshikawa, Phys. Rev. Lett. **114**, 251603 (2015).
[S5] D. V. Else, B. Bauer, and C. Nayak, Phys. Rev. Lett. **117**, 090402 (2016).
[S6] N. Y. Yao, A. C. Potter, I.-D. Potirniche, and A. Vishwanath, Phys. Rev. Lett. **118**, 030401 (2017).
[S7] V. Khemani, A. Lazarides, R. Moessner, and S. L. Sondhi, Phys. Rev. Lett. **116**, 250401 (2016).
[S8] Y. Bahri, R. Vosk, E. Altman, and A. Vishwanath, Nat. Commun. **6**, 7341 (2015).
[S9] M. Serbyn, Z. Papić, and D. A. Abanin, Phys. Rev. Lett **111**, 127201 (2013).
[S10] J. H. Bardarson, F. Pollmann, and J. E. Moore, Phys. Rev. Lett. **109**, 017202 (2012).
[S11] C. W. von Keyserlingk, V. Khemani, and S. L. Sondhi, Phys. Rev. B **94**, 085112 (2016).
[S12] D. A. Abanin, E. Altman, I. Bloch, and M. Serbyn, Rev. Mod. Phys. **91**, 021001 (2019).
[S13] A. Kumar, P. T. Dumitrescu, and A. C. Potter, Phys. Rev. B **97**, 224302 (2018).
[S14] A. Y. Kitaev, Physics-Uspekhi **44**, 131 (2001).
[S15] C. W. von Keyserlingk and S. L. Sondhi, Phys. Rev. B **93**, 245145 (2016).
[S16] G. Vidal, Phys. Rev. Lett. **91**, 147902 (2003).

[S17] G. Vidal, Phys. Rev. Lett. **93**, 040502 (2004).
[S18] S. R. White and A. E. Feiguin, Phys. Rev. Lett. **93**, 076401 (2004).
[S19] A. J. Daley, C. Kollath, U. Schollwöck, and G. Vidal, J. Stat. Mech.: Theor. Exp. **2004**, P04005 (2004).
[S20] A. Peruzzo, J. McClean, P. Shadbolt, M.-H. Yung, X.-Q. Zhou, P. J. Love, A. Aspuru-Guzik, and J. L. O'Brien, Nat Commun. **5**, 4213 (2014).
[S21] K. Beer, D. Bondarenko, T. Farrelly, T. J. Osborne, R. Salzmann, D. Scheiermann, and R. Wolf, Nat. Commun. **11**, 808 (2020).
[S22] Z. Lu, P.-X. Shen, and D.-L. Deng, Phys. Rev. Appl. **16**, 044039 (2021).
[S23] X. Wang, Z. Sun, and Z. D. Wang, Phys. Rev. A **79**, 012105 (2009).
[S24] F. Yan, P. Krantz, Y. Sung, M. Kjaergaard, D. L. Campbell, T. P. Orlando, S. Gustavsson, and W. D. Oliver, Phys. Rev. Appl. **10**, 054062 (2018).
[S25] K. Zhang, H. Li, P. Zhang, J. Yuan, J. Chen, W. Ren, Z. Wang, C. Song, D.-W. Wang, H. Wang, S. Zhu, G. S. Agarwal, and M. O. Scully, arXiv:2109.00964 (2021).
[S26] D. C. McKay, C. J. Wood, S. Sheldon, J. M. Chow, and J. M. Gambetta, Phys. Rev. A **96**, 022330 (2017).
[S27] Z. Wang, H. Li, W. Feng, X. Song, C. Song, W. Liu, Q. Guo, X. Zhang, H. Dong, D. Zheng, H. Wang, and D.-W. Wang, Phys. Rev. Lett. **124**, 013601 (2020).
[S28] P. V. Klimov, J. Kelly, Z. Chen, M. Neeley, A. Megrant, B. Burkett, R. Barends, K. Arya, B. Chiaro, Y. Chen, A. Dunsworth, A. Fowler, B. Foxen, C. Gidney, M. Giustina, R. Graff, T. Huang, E. Jeffrey, E. Lucero, J. Y. Mutus, O. Naaman, C. Neill, C. Quintana, P. Roushan, D. Sank, A. Vainsencher, J. Wenner, T. C. White, S. Boixo, R. Babbush, V. N. Smelyanskiy, H. Neven, and J. M. Martinis, Phys. Rev. Lett. **121**, 090502 (2018).
[S29] W. Ren, W. Liu, C. Song, H. Li, Q. Guo, Z. Wang, D. Zheng, G. S. Agarwal, M. O. Scully, S.-Y. Zhu, H. Wang, and D.-W. Wang, Phys. Rev. Lett. **125**, 133601 (2020).
[S30] C. Song, K. Xu, W. Liu, C.-p. Yang, S.-B. Zheng, H. Deng, Q. Xie, K. Huang, Q. Guo, L. Zhang, P. Zhang, D. Xu, D. Zheng, X. Zhu, H. Wang, Y.-A. Chen, C.-Y. Lu, S. Han, and J.-W. Pan, Phys. Rev. Lett. **119**, 180511 (2017).
[S31] Y. Xu, J. Chu, J. Yuan, J. Qiu, Y. Zhou, L. Zhang, X. Tan, Y. Yu, S. Liu, J. Li, F. Yan, and D. Yu, Phys. Rev. Lett. **125**, 240503 (2020).
[S32] M. A. Rol, F. Battistel, F. K. Malinowski, C. C. Bultink, B. M. Tarasinski, R. Vollmer, N. Haider, N. Muthusubramanian, A. Bruno, B. M. Terhal, and L. DiCarlo, Phys. Rev. Lett. **123**, 120502 (2019).
[S33] M. Cheneau, P. Barmettler, D. Poletti, M. Endres, P. Schauß, T. Fukuhara, C. Gross, I. Bloch, C. Kollath, and S. Kuhr, Nature **481**, 484 (2012).
[S34] P. Richerme, Z.-X. Gong, A. Lee, C. Senko, J. Smith, M. Foss-Feig, S. Michalakis, A. V. Gorshkov, and C. Monroe, Nature **511**, 198 (2014).
[S35] P. Jurcevic, B. Lanyon, P. Hauke, C. Hempel, P. Zoller, R. Blatt, C. Roos, *et al.*, Nature **511**, 202 (2014).
[S36] V. Khemani, R. Moessner, and S. L. Sondhi, "A Brief History of Time Crystals," (2019), arXiv:1910.10745.
[S37] D. V. Else and C. Nayak, Phys. Rev. B **93**, 201103 (2016).
[S38] I.-D. Potirniche, A. C. Potter, M. Schleier-Smith, A. Vishwanath, and N. Y. Yao, Phys. Rev. Lett. **119**, 123601 (2017).
[S39] A. C. Potter, T. Morimoto, and A. Vishwanath, Phys. Rev. X **6**, 041001 (2016).
[S40] A. J. Friedman, B. Ware, R. Vasseur, and A. C. Potter, Phys. Rev. B **105**, 115117 (2022).
[S41] P. T. Dumitrescu, J. Bohnet, J. Gaebler, A. Hankin, D. Hayes, A. Kumar, B. Neyenhuis, R. Vasseur, and A. C. Potter, arXiv:2107.09676 (2021).
